# Supplementary material for: MAAT: a new nonparametric Bayesian framework for incorporating multiple functional annotations in transcriptome-wide association studies
Source: Genome Biol. 2025 Feb 4;26:21. doi: 10.1186/s13059-025-03485-x (PMC11796105; doi:10.1186/s13059-025-03485-x)
Supplement: Supplementary file 1 — Additional file 1: Supplementary Materials that include additional methods, tables and figures. [file 13059_2025_3485_MOESM1_ESM.pdf]

Supplementary Note for MAAT: a new nonparametric Bayesian framework for incorporating multiple functional annotations in transcriptome-wide association studies

1 The complete PPMx model in MAAT

In the imputation step, for each gene g, we consider a linear relationship between its genotype information and gene expression profiles:

$$\boldsymbol{E}_g = \boldsymbol{X}\beta + \boldsymbol{\epsilon} = \boldsymbol{X}\tilde{\boldsymbol{\beta}} + \boldsymbol{u} + \boldsymbol{\epsilon}, \quad \boldsymbol{\epsilon} \sim N(0, \sigma_e^2 \mathbf{I}_n),$$

where $\boldsymbol{E}_g$ is an n-vector denoting the expression profiles of gene g across n individuals, $\boldsymbol{X}$ is a gene-specific $n \times p$ genotype matrix, where each column contains the genotype information for a cis-SNP of gene g. $\boldsymbol{\beta}$ is the effect size vector, and $\boldsymbol{\epsilon}$ is the residual error, with each element following a Gaussian distribution with mean 0 and variance σ_e^2. $\boldsymbol{u} \sim N(\mathbf{0}, \sigma_e^2\sigma_0^2\boldsymbol{K})$ is the random effect term introduced to simplify computation [1, 2, 3], where $\boldsymbol{K} = \boldsymbol{X}\boldsymbol{X}^\top/p$ is the genetic relatedness matrix (GRM). Therefore, $\boldsymbol{u}$ can also be represented as:

$$\boldsymbol{u} = \boldsymbol{X}\boldsymbol{\xi}, \ \boldsymbol{\xi} \sim N\left(0, \frac{\sigma_e^2\sigma_0^2}{p}\mathbf{I}_p\right).$$

We drop the intercept term for assuming $\boldsymbol{E}_g$ and $\boldsymbol{X}$ are standardized.

We assume $\rho = \{S_1, S_2, \ldots, S_{k_p}\}$ is a partition on p cis-SNPs, with S_j being the j-th cluster containing the index of cis-SNPs belonging to it. Correspondingly, we have $\boldsymbol{Z} = (z_1, \ldots, z_p)$ denoting the cluster index for p cis-SNPs, i.e., $z_k = j$ or $k \in S_j$ if the k-th cis-SNP belongs to cluster j under partition ρ. As described in the main text, suppose there are m annotation scores allocated to cis-SNP k, which are denoted as

$\boldsymbol{W}_k = (W_{k1}, \cdots, W_{km})$. The annotation matrix for all p cis-SNPs can be represented by $\boldsymbol{W} = (\boldsymbol{W}_1^T, \cdots, \boldsymbol{W}_p^T) \in \mathbb{R}^{m \times p}$. Let $\boldsymbol{W}_j^* = \{\boldsymbol{W}_k : k \in S_j\} \in \mathbb{R}^{m \times |S_j|}$ denote the cluster j-specific annotation information, and let $\boldsymbol{W}_{jl}^*$, the l-th row of $\boldsymbol{W}_j^*$, denote a $|S_j|$-vector containing the l-th annotation information for cis-SNPs in cluster j, where $|S_j|$ is the cardinality of set S_j. The complete PPMx model [4] in MAAT is:

$$
\begin{aligned}
&\boldsymbol{E}_g = \boldsymbol{X}\boldsymbol{\beta} + \boldsymbol{\epsilon} = \boldsymbol{X}\tilde{\boldsymbol{\beta}} + \boldsymbol{u} + \boldsymbol{\epsilon};\ \boldsymbol{\epsilon} \sim N(0, \sigma_e^2 \boldsymbol{I}_n) \\
&\boldsymbol{u} = \boldsymbol{X}\boldsymbol{\xi},\ \xi_k \sim N(0, \sigma_e^2\sigma_0^2/p),\ k = 1, \cdots, p \\
&\tilde{\beta}_k | z_k = j, \sigma_e^2, \sigma_j^2 \sim N(0, \sigma_e^2\sigma_j^2),\ \text{for } k = 1, \cdots, p; \\
&\sigma_e^2 \sim \text{IG}(a_e, b_e);\ \sigma_0^2 \sim \text{IG}(a, b);\ \sigma_j^2 \sim \text{IG}(a, b),\ \text{for } j = 1, \cdots, k_p; \\
&P(\boldsymbol{Z}|\boldsymbol{W}) \propto \prod_{j=1}^{k_p} g(\boldsymbol{W}_j^*) C(S_j) \\
&g(\boldsymbol{W}_j^*) = \prod_{l=1}^{m} g(\boldsymbol{W}_{jl}^*);\quad g(\boldsymbol{W}_{jl}^*) = \prod_{t \in S_j} \phi(W_{tl} | \hat{\mu}_{jl}, \hat{\sigma}_{jl}^2) \\
&C(S_j) = (|S_j| - 1)!
\end{aligned}
\tag{1.1}
$$

where $\text{IG}(a, b)$ is the inverse gamma distribution with shape parameter a and scale parameter b. $C(S_j)$ is the cohesion function to prevent similar cis-SNPs from being splitted into small clusters. $g(\boldsymbol{W}_j^*)$ is a similarity function indicating the homogeneity level of cis-SNPs in cluster j. The higher homogeneity level of cis-SNPs in cluster j, the larger $g(\boldsymbol{W}_j^*)$ is [5]. $\phi(x|\mu, \sigma^2)$ is the density function of a normal distribution with mean μ and variance σ^2. $\hat{\mu}_{jl}$ and $\hat{\sigma}_{jl}^2$ are the sample mean and sample variance of $\boldsymbol{W}_{jl}^*$.

In our MAAT method, the similarity function is a particular choice of double dipping similarity function [6]:

$$
g(\boldsymbol{W}_{jl}^*) = \int \prod_{t \in S_j} q_1(W_{tl} | \boldsymbol{\xi}_{jl}^*) q_2(\boldsymbol{\xi}_{jl}^* | \boldsymbol{W}_{jl}^*) d\boldsymbol{\xi}_{jl}^* \tag{1.2}
$$

where $q_1(W_{tl}|\boldsymbol{\xi}_{jl}^*)$ and $q_2(\boldsymbol{\xi}_{jl}^*|\boldsymbol{W}_{jl}^*)$ are density functions. $\boldsymbol{\xi}_{jl}^*$ is a vector containing parameters in density function $q(W_{tl}|\boldsymbol{\xi}_{jl}^*)$. $q_2(\boldsymbol{\xi}_{jl}^*|\boldsymbol{W}_{jl}^*)$ can be viewed as a "posterior" distribution for $\boldsymbol{\xi}_{jl}^*$ given annotation information $\boldsymbol{W}_{jl}^*$. For computational simplicity, we let $q_1(W_{tl}|\boldsymbol{\xi}_{jl}^*) = \phi(W_{tl}|\mu_{jl}, \sigma_{jl}^2)$ and $\boldsymbol{\xi}_{jl}^* = (\mu_{jl}, \sigma_{jl}^2)$ in this case. We further let $q_2(\boldsymbol{\xi}_{jl}^*|\boldsymbol{W}_{jl}^*)$ be the density function corresponding to a point mass distribution at $(\hat{\mu}_{jl}, \hat{\sigma}_{jl}^2)$, where $\hat{\mu}_{jl}$ and $\hat{\sigma}_{jl}^2$ are the sample mean and sample variance of $\boldsymbol{W}_{jl}^*$. Specifically,

$$
q_2(\boldsymbol{\xi}_{jl}^*|\boldsymbol{W}_{jl}^*) = \begin{cases} 1, & \boldsymbol{\xi}_{jl}^* = (\mu_{jl}, \sigma_{jl}^2) = (\hat{\mu}_{jl}, \hat{\sigma}_{jl}^2) \\ 0, & \boldsymbol{\xi}_{jl}^* = (\mu_{jl}, \sigma_{jl}^2) \neq (\hat{\mu}_{jl}, \hat{\sigma}_{jl}^2) \end{cases}
$$

Therefore, based on the choice of $q_1(W_{tl}|\boldsymbol{\xi}^*_{jl})$ and $q_2(\boldsymbol{\xi}^*_{jl}|\boldsymbol{W}^*_{jl})$, the similarity function $g(\boldsymbol{W}^*_{jl})$ reaches to the form in (1.1).

Then the joint conditional posterior function is:

$$
\begin{aligned}
&P(\tilde{\boldsymbol{\beta}}, \boldsymbol{u}, \boldsymbol{Z}, \sigma_e^2, \sigma_0^2, \sigma_1^2, \cdots, \sigma_{k_p}^2 | \boldsymbol{E}_g, \boldsymbol{X}, \boldsymbol{W}) \propto P(\boldsymbol{E}_g|\boldsymbol{X}, \boldsymbol{u}, \tilde{\boldsymbol{\beta}}, \sigma_e^2) P(\tilde{\boldsymbol{\beta}}|\boldsymbol{Z}, \sigma_1^2, \cdots, \sigma_{k_p}^2, \sigma_e^2) \\
&P(\boldsymbol{u}|\sigma_e^2, \sigma_0^2, \boldsymbol{X}) P(\boldsymbol{Z}|\boldsymbol{W}) \prod_{j=0}^{k_p} P(\sigma_j^2|a, b) P(\sigma_e^2|a_e, b_e) \qquad (1.3)
\end{aligned}
$$

After integrating out $\boldsymbol{u}$ in (1.3), the log joint conditional posterior density function is:

$$
\begin{aligned}
&\log P(\tilde{\boldsymbol{\beta}}, \boldsymbol{Z}, \sigma_e^2, \sigma_0^2, \sigma_1^2, \cdots, \sigma_{k_p}^2 | \boldsymbol{E}_g, \boldsymbol{X}, \boldsymbol{W}) \\
=&C - \frac{1}{2}\log|\sigma_e^2 \boldsymbol{H}| - \frac{1}{2\sigma_e^2}(\boldsymbol{E}_g - \boldsymbol{X}\tilde{\boldsymbol{\beta}})^\top \boldsymbol{H}^{-1}(\boldsymbol{E}_g - \boldsymbol{X}\tilde{\boldsymbol{\beta}}) + \sum_{k=1}^{p}\left(-\frac{1}{2}\log(\sigma_e^2\sigma_{z_k}^2) - \frac{\tilde{\beta}_k^2}{2\sigma_e^2\sigma_{z_k}^2}\right) \\
&-\sum_{j=0}^{k_p}\left((a+1)\log(\sigma_j^2) + \frac{b}{\sigma_j^2}\right) - (a_e+1)\log(\sigma_e^2) - \frac{b_e}{\sigma_e} \qquad (1.4) \\
&+\sum_{j=1}^{k_p}\log\left((|S_j|-1)!\right) + \sum_{j=1}^{k_p}\sum_{l=1}^{m}\sum_{t\in S_j}\left(-\frac{1}{2}\log(\hat{\sigma}_{jl}^2) - \frac{(W_{tl}-\hat{\mu}_{tl})^2}{2\hat{\sigma}_{jl}^2}\right)
\end{aligned}
$$

where $\boldsymbol{H} = \boldsymbol{I}_n + \sigma_0^2\boldsymbol{K}$ and $\boldsymbol{K} = \boldsymbol{X}\boldsymbol{X}^\top/p$. In practice, we set a, b, a_e and b_e to be 0.1.

2 Some additional notes on the PPMx model

To enhance the clarity of PPMx adopted in MAAT, we provide two additional explanations:

- Introducing a prior to $\boldsymbol{Z}$ is equivalent to imposing a discrete prior defined over p^p potential outcomes, where p is the number of cis-SNPs. Each outcome of $\boldsymbol{Z}$ corresponds to a specific clustering of the p cis-SNPs. The annotation likelihood on this clustering regime is proportional to the prior probability of the corresponding outcome of $\boldsymbol{Z}$. Consequently, if a clustering regime on the cis-SNPs' annotation profile is rational, it leads to a higher annotation likelihood and subsequently a higher probability to the corresponding outcome of $\boldsymbol{Z}$ can be assigned.

- The introduction of the cohesion function ensures that cis-SNPs with homogeneous annotation profiles can be grouped together, preventing them from being

fragmented into numerous smaller clusters. For instance, if all 1000 cis-SNPs of gene g share highly similar annotation profiles, the prior probability of treating each cis-SNP itself as a cluster is $P(\boldsymbol{Z}|\boldsymbol{W}) = \prod_{j=1}^{1000} g(\boldsymbol{W}_j^*)$, where $\boldsymbol{Z} = (1, \ldots, 1000)$. In contrast, the prior probability of consolidating all 1000 cis-SNPs into a single cluster is $P(\boldsymbol{Z}|\boldsymbol{W}) = 999!g(\boldsymbol{W}_1^*)$, where $\boldsymbol{Z} = (1, 1, \ldots, 1)$. It is evident that this prior probability is much larger than the probability imposed on $\boldsymbol{Z} = (1, \ldots, 1000)$. Therefore, with the incorporation of the cohesion function, in cases where cis-SNPs exhibit similar annotations, MAAT is more inclined to group them into a unified cluster rather than fragmenting them into numerous clusters.

3 MCMC algorithm

3.1 update $\tilde{\beta}_k$

The log conditional density function for $\tilde{\beta}_k$ is given by

$$\log p(\tilde{\beta}_k|\cdot) = C + \frac{\tilde{\beta}_k^2}{2\sigma_e^2}(\sigma_{z_k}^{-2} + \boldsymbol{x}_k'\boldsymbol{H}^{-1}\boldsymbol{x}_k) + \frac{\tilde{\beta}_k}{\sigma_e^2}\left[\boldsymbol{x}_k'\boldsymbol{H}^{-1}\boldsymbol{E}_g - \sum_{j\neq k}\boldsymbol{x}_k'\boldsymbol{H}^{-1}\boldsymbol{x}_j\tilde{\beta}_j\right]$$

where C is a constant irrelevant to $\tilde{\beta}_k$. Therefore, the conditional distribution for sampling $\tilde{\beta}_k$ is:

$$\tilde{\beta}_k \sim N\left(\frac{\boldsymbol{x}_k'\boldsymbol{H}^{-1}\boldsymbol{E}_g - \sum_{j\neq k}\boldsymbol{x}_k'\boldsymbol{H}^{-1}\boldsymbol{x}_j\tilde{\beta}_j}{\sigma_{z_k}^{-2} + \boldsymbol{x}_k'\boldsymbol{H}^{-1}\boldsymbol{x}_k}, \frac{\sigma_e^2}{\sigma_{z_k}^{-2} + \boldsymbol{x}_k'\boldsymbol{H}^{-1}\boldsymbol{x}_k}\right)$$

3.2 update σ_j^2

The conditional density function for σ_j^2 is given by

$$p(\sigma_j^2|\cdot) \propto \left[\prod_{z_k=j}\frac{1}{\sqrt{2\pi\sigma_e^2\sigma_j^2}}\exp\left\{-\frac{\tilde{\beta}_k^2}{2\sigma_e^2\sigma_j^2}\right\}\right]\frac{b^a}{\Gamma(a)}\left(\frac{1}{\sigma_j^2}\right)^{a+1}\exp\left\{-\frac{b}{\sigma_j^2}\right\}$$

If we let $n_j = \#\{k|z_k = j, k = 1, \ldots, p\}$, i.e., n_j is the number of cis-SNPs which belongs to cluster j, then the conditional distribution for sampling σ_j^2 is:

$$\sigma_j^2 \sim IG\left(\frac{n_j}{2} + a, \frac{\sum_{z_k=j}\tilde{\beta}_k^2}{2\sigma_e^2} + b\right), \ j = 1, \cdots, k_p$$

3.3 update z_k

Following [7] and [5], we have

$$p(z_k = j|\cdot) \propto \begin{cases} \phi(\tilde{\beta}_k|0, \sigma_e^2\sigma_j^2) \dfrac{C(S_j^{-k} \bigcup \{k\}) g(\boldsymbol{W}_j^{*-k} \bigcup \{\boldsymbol{W}_k\})}{C(S_j^{-k}) g(\boldsymbol{W}_j^{*-k})} & \text{if } j = 1, \ldots, p^{-k} \\ \phi(\tilde{\beta}_k|0, \sigma_e^2\sigma_j^2) C(\{k\}) g(\boldsymbol{W}_k) & \text{if } j = p^{-k} + 1 \end{cases}$$

where p^{-k} is the number of clusters when SNP k is not considered. $p^{-k} = k_p$ if cis-SNP k belongs to a cluster which has more than two cis-SNPs, and $p^{-k} = k_p - 1$ if cis-SNP k itself forms a cluster. S_j^{-k} is the j-th cluster when cis-SNP k is not considered. $\boldsymbol{W}_j^* = \{\boldsymbol{W}_k : k \in S_j\}$ denote the cluster j-specific annotation information, $\boldsymbol{W}_j^{*-k}$ is the annotation information matrix for cluster j when cis-SNP k is not considered, and $\boldsymbol{W}_k$ is the annotation vector for cis-SNP k.

In practice, we set k_p no more than 10 to reduce computational time.

3.4 update σ_e^2

The conditional density function for σ_e^2 is given by

$$p(\sigma_e^2|\cdot) \propto \left(\frac{1}{\sigma_e^2}\right)^{\frac{n}{2}+a_e+\frac{p}{2}} \exp\left[-\frac{1}{\sigma_e^2}\left\{\frac{1}{2}(\boldsymbol{Y} - \boldsymbol{X}\tilde{\boldsymbol{\beta}})^\top H^{-1}(\boldsymbol{Y} - \boldsymbol{X}\tilde{\boldsymbol{\beta}}) + b_e + \frac{1}{2}\sum_{j=1}^{k_p}\left(\sum_{z_k=j}\tilde{\beta}_k^2/\sigma_j^2\right)\right\}\right]$$

Therefore, the conditional distribution for sampling σ_e^2 is

$$\sigma_e^2 \sim IG\left(\frac{n}{2} + a_e + \frac{p}{2}, \frac{1}{2}(\boldsymbol{Y} - \boldsymbol{X}\tilde{\boldsymbol{\beta}})^\top H^{-1}(\boldsymbol{Y} - \boldsymbol{X}\tilde{\boldsymbol{\beta}}) + b_e + \frac{1}{2}\sum_{j=1}^{k_p}\left(\sum_{z_k=j}\tilde{\beta}_k^2/\sigma_j^2\right)\right)$$

3.5 update σ_0^2

The conditional density function for σ_0^2 is given by

$$f(\sigma_0^2|\cdot) \propto \frac{1}{|\boldsymbol{H}|^{\frac{1}{2}}} \exp\left\{-\frac{1}{2\sigma_e^2}(\boldsymbol{Y} - \boldsymbol{X}\tilde{\boldsymbol{\beta}})^\top \boldsymbol{H}^{-1}(\boldsymbol{Y} - \boldsymbol{X}\tilde{\boldsymbol{\beta}})\right\}\left(\frac{1}{\sigma_0^2}\right)^{a+1} \exp\left\{-\frac{b}{\sigma_0^2}\right\}$$

Following [1, 2], and [8], we adopt reparameterization of σ_0^2 to conduct sampling. Specifically, let $h^2 = \sigma_0^2/1 + \sigma_0^2$, then we have

$$f(h^2|\cdot) \propto \frac{1}{|\boldsymbol{H}|^{\frac{1}{2}}} \exp\left\{-\frac{1}{2\sigma_e^2}(\boldsymbol{Y} - \boldsymbol{X}\tilde{\boldsymbol{\beta}})^\top \boldsymbol{H}^{-1}(\boldsymbol{Y} - \boldsymbol{X}\tilde{\boldsymbol{\beta}}) - \frac{b(1-h^2)}{h^2}\right\}\left(\frac{1-h^2}{h^2}\right)^{a+1}\left(\frac{1}{1-h^2}\right)^2$$

We use the Metropolis-Hastings algorithm to generate h^2, with the proposal distribution $Q(y|x) \equiv Q(y) = Beta(y; a_\beta, b_\beta)$, and we set $a_\beta = 2$, $b_\beta = 8$ here.

In the i-th iteration, the acceptance ratio for determining whether to transport from h_{i-1}^2 in the last iteration to h_i^2 is:

$$\min\left\{\frac{f(h_1^2)Q(h_0^2)}{f(h_0^2)Q(h_1^2)}, 1\right\}$$

4 Annotation assignment for genes

For GWAS, we conduct the per-SNP association analysis by fitting the following one-SNP-at-a-time linear regression:

$$\boldsymbol{Y} = \boldsymbol{X}_j \alpha_j + \boldsymbol{\epsilon}_j,\ \boldsymbol{\epsilon}_j \sim N(\boldsymbol{0}, \sigma_j^2 \boldsymbol{I}_n)$$

where $\boldsymbol{Y}$ is a standardized n-vector of phenotyes, $\boldsymbol{X}_j$ is a standardized n-vector denoting genotype information for SNP j, α_j is the marginal effect size of SNP j and $\boldsymbol{\epsilon}_j$ is an n-vector of residual errors with each element following a Gaussian distribution. We can obtain $\hat{\alpha}_j = \boldsymbol{X}_j' \boldsymbol{Y}$ and $\mathrm{Var}(\hat{\alpha}_j) = \sigma_j^2$ by calculation. In practice, σ_j^2 is unknown, we estimate it by:

$$\hat{\sigma}_j^2 = \frac{\hat{\boldsymbol{\epsilon}_j}^\top \hat{\boldsymbol{\epsilon}_j}}{n-2} = \frac{(\boldsymbol{Y} - \boldsymbol{X}_j\hat{\alpha}_j)^\top (\boldsymbol{Y} - \boldsymbol{X}_j\hat{\alpha}_j)}{n-2} = \frac{\boldsymbol{Y}'(\boldsymbol{I}_n - \boldsymbol{X}_j\boldsymbol{X}_j^\top)\boldsymbol{Y}}{n-2} = \frac{1-(\boldsymbol{X}_j'\boldsymbol{Y})^2}{n-2}$$

Thus based on the first-order Taylor expansion, the Z-score for SNP j can be approximated by $Z_j = \hat{\alpha}_j / se(\hat{\alpha}_j) \approx \sqrt{n-2}\boldsymbol{X}_j'\boldsymbol{Y}$. We assume $\boldsymbol{Z}_{SG} = (Z_1, \cdots, Z_p)^\top$ is a p-vector containing GWAS Z-scores for p cis-SNPs. Let $\boldsymbol{X} = (\boldsymbol{X}_1, \cdots, \boldsymbol{X}_p)$ denote the gene-specific $n \times p$ genotype matrix, we can also fit a joint model:

$$\boldsymbol{Y} = \boldsymbol{X}\tilde{\boldsymbol{\alpha}} + \boldsymbol{\epsilon},\ \boldsymbol{\epsilon} \sim N(\boldsymbol{0}, \sigma^2 \boldsymbol{I}_n)$$

where $\tilde{\boldsymbol{\alpha}}$ is the joint effect size for all p cis-SNPs. Then we have:

$$E\boldsymbol{Z}_{SG} \approx \sqrt{n-2}E(\boldsymbol{X}_1'\boldsymbol{Y}, \cdots, \boldsymbol{X}_p'\boldsymbol{Y})^\top = \sqrt{n-2}E(\boldsymbol{X}'\boldsymbol{Y}) = \sqrt{n-2}\boldsymbol{X}'\boldsymbol{X}\tilde{\boldsymbol{\alpha}}$$

For the TWAS test statistic, we can derive its mean:

$$EZ = \frac{\boldsymbol{\beta}'}{\sqrt{\boldsymbol{\beta}'\boldsymbol{V}\boldsymbol{\beta}}} E\boldsymbol{Z}_{SG} \approx \sqrt{n-2}\frac{\boldsymbol{\beta}'\boldsymbol{V}\tilde{\boldsymbol{\alpha}}}{\sqrt{\boldsymbol{\beta}'\boldsymbol{V}\boldsymbol{\beta}}} \tag{4.1}$$

where $\boldsymbol{\beta}$ is the cis-SNPs' effect size on the expression level of a specific gene g, $\boldsymbol{V}$ is the covariance matrix for all cis-SNPs of gene g. If we perform an eigendecomposition on $\boldsymbol{V}$, i.e. $\boldsymbol{V} = \boldsymbol{U}\boldsymbol{\Lambda}\boldsymbol{U}'$, and we let $\boldsymbol{\beta}_r = \boldsymbol{\Lambda}^{\frac{1}{2}}\boldsymbol{U}'\boldsymbol{\beta}$ and $\tilde{\boldsymbol{\alpha}}_r = \boldsymbol{\Lambda}^{\frac{1}{2}}\boldsymbol{U}'\tilde{\boldsymbol{\alpha}}$ denote the rotated effect sizes, (4.1) can be transformed into:

$$EZ \approx \sqrt{n-2}||\tilde{\boldsymbol{\alpha}}_r|| \cos(\boldsymbol{\beta}_r, \tilde{\boldsymbol{\alpha}}_r) \tag{4.2}$$

Based on (4.2), we can reach the conclusion that the power of TWAS is determined by the angle between the rotated cis-SNPs' effect size on gene expression and phenotype. In practice, the joint effect size $\boldsymbol{\alpha}$ is hard to obtain, so we use marginal effect size from GWAS summary data to approximate the joint effect size. Integrated with the evidence that SNPs associated with complex traits are more likely to be eQTL [9, 10], the angle between $\boldsymbol{\beta}_r$ and $\tilde{\boldsymbol{\alpha}}_r$ is guaranteed to be not very small for TWAS, compared with the classical burden-type test statistic whose $\boldsymbol{\beta} = \mathbf{1}_p/\sqrt{p}$. Thus by (4.2), we can establish a statistical framework to explain why TWAS behaves better than burden test for gene-based association analysis.

When switching to annotation-assisted TWAS, if there are m annotations allocated to each SNP, we let $\tilde{\boldsymbol{W}}_l = (W_{1l}, \ldots, W_{pl})^\top$ denote the l-th annotation information in all p cis-SNPs for $l = 1, \ldots, m$. Similar as before, we define $\tilde{\boldsymbol{W}}_{l,r} = \boldsymbol{\Lambda}^{\frac{1}{2}}\boldsymbol{U}'\tilde{\boldsymbol{W}}_l$ as the rotated annotation. We treat annotation l as an important annotation if the two cosine distances $\cos(\tilde{\boldsymbol{W}}_{l,r}, \boldsymbol{\beta}_r)$ and $\cos(\tilde{\boldsymbol{W}}_{l,r}, \tilde{\boldsymbol{\alpha}}_r)$ are both large compared with other annotations. Therefore, we assign important annotation l^* to each gene by the following fomula:

$$l^* = \underset{l=1,\cdots,L}{\operatorname{argmax}} \left| \cos(\tilde{\boldsymbol{W}}_{l,r}, \boldsymbol{\beta}_r) + \cos(\tilde{\boldsymbol{W}}_{l,r}, \boldsymbol{\alpha}_r) \right|,$$

where L is the number of annotations included in MAAT. In this study, we set $L = 7$.

Suppose the effect size obtained from the first imputation step of MAAT is $\boldsymbol{\beta}^M$, the burder-type effect size is $\boldsymbol{\beta}^B$, their corresponding rotated effect sizes are $\boldsymbol{\beta}_r^M$ and $\boldsymbol{\beta}_r^B$. We can interpret the role which annotation information plays in TWAS as follows: when $\cos(\tilde{\boldsymbol{W}}_{l,r}, \tilde{\boldsymbol{\alpha}}_r)$ is large, the SNPs' contribution to the phenotype can be well reflected by the l-th annotation score; while the prior in MAAT drag the effect size towards the direction of $\tilde{\boldsymbol{W}}_{l,r}$, which is near to the direction of GWAS effect sizes, resulting in the enhanced power of TWAS.

5 Post-processing of β

We implement a post-processing step on β to achieve refine accuracy. Specifically, utilizing the PPMx model, cis-SNPs are partitioned into k_p groups. Cis-SNPs within groups demonstrating higher effect sizes (indicated by higher σ_j^2 for the j-th group) are considered as more important ones. For each gene, we run 20,000 MCMC iterations. The

initial 10,000 iterations serve as burn in, while the remaining 10,000 iterations are reserved for parameter estimation. For the i-th iteration, we can rank the importance of cluster j based on its corresponding σ_j^2. If the k-th SNP falls into the top 20% of unimportant clusters, (i.e., the cluster j to which SNP i belongs has an σ_j^2 ranking among the smallest 20% of all $\sigma_1^2, \ldots, \sigma_{k_p}^2$ in the i-th MCMC iteration), we categorize this k-th SNP as unimportant. If we introduce r_{ki} to signify whether SNP k is important in the i-th iteration, with $r_{ki} = 0$ indicating unimportance and $r_{ki} = 1$ indicating importance. Therefore, for each SNP k, we calculate

$$r_k = \sum_{i=10001}^{20000} r_{ki}$$

to denote the importance level for SNP k. Based on the vector $\boldsymbol{r} = (r_1, \ldots, r_p)$, we can post-process β into different sparsity levels. For instance, achieving 10% sparsity level involves setting the coefficient of 90% of SNPs to 0, where the r_k values of these SNPs rank among the lowest 90% among all SNPs.

6 Pathway enrichment analysis after colocalization

To assess the impact of integrating TWAS with colocalization analysis, for each psychiatric trait, we examined the overlap between TWAS significant genes and the gene set with gene-level colocalization probability (GLCP) larger than 0.001, and then conducted pathway enrichment analysis. As shown in Fig. S41–48, for most psychiatric traits, the pathway enrichment patterns remain largely consistent with with those shown in Fig. S32–39. after colocalization post-processing. It is worth noting that, several key pathways demonstrate increased enrichment level, indicating that colocalization analysis can effectively filter out some TWAS false positives. For example, the enrichment level of response to osmotic stress pathway in Alzheimer's disease (AD) has increased [11]. The ranking of mitochondrial matrix pathway has improved for insomnia, consistent with previous research suggesting a link between insomnia and mitochondrial dysfunction [12]. Similarly, spinocerebellar ataxia (SCA) and the presynaptic membrane pathway have a higher enrichment rank in bipolar disorder [13, 14]. However, some critical pathways have become non-significant due to the low GLCP levels of some TWAS significant genes. These include the congenital malformations of the nervous system pathway in anorexia nervosa, the Parkinson's disease pathway in Parkinson's disease (PD), and the dendritic

spine membrane pathway in schizophrenia, which underscore a complementary nature between TWAS and colocalization analyses. Therefore, as discussed in the main text, integrating TWAS fine-mapping tools with colocalization methods offers a promising approach to refining TWAS results and enhancing their interpretability and reliability.

References

[1] Xiang Zhou, Peter Carbonetto, and Matthew Stephens. Polygenic modeling with bayesian sparse linear mixed models. *PLoS genetics*, 9(2):e1003264, 2013.

[2] Ping Zeng and Xiang Zhou. Non-parametric genetic prediction of complex traits with latent dirichlet process regression models. *Nature communications*, 8(1):1–11, 2017.

[3] Sini Nagpal, Xiaoran Meng, Michael P Epstein, Lam C Tsoi, Matthew Patrick, Greg Gibson, Philip L De Jager, David A Bennett, Aliza P Wingo, Thomas S Wingo, et al. Tigar: an improved bayesian tool for transcriptomic data imputation enhances gene mapping of complex traits. *The American Journal of Human Genetics*, 105(2):258–266, 2019.

[4] Peter Müller, Fernando Quintana, and Gary L Rosner. A product partition model with regression on covariates. *Journal of Computational and Graphical Statistics*, 20(1):260–278, 2011.

[5] Garritt L Page and Fernando A Quintana. Calibrating covariate informed product partition models. *Statistics and Computing*, 28(5):1009–1031, 2018.

[6] Fernando A Quintana, Peter Müller, and Ana Luisa Papoila. Cluster-specific variable selection for product partition models. *Scandinavian Journal of Statistics*, 42(4):1065–1077, 2015.

[7] Radford M Neal. Markov chain sampling methods for dirichlet process mixture models. *Journal of computational and graphical statistics*, 9(2):249–265, 2000.

[8] Andrew Gelman. Parameterization and bayesian modeling. *Journal of the American Statistical Association*, 99(466):537–545, 2004.

[9] Frank W Albert and Leonid Kruglyak. The role of regulatory variation in complex traits and disease. *Nature Reviews Genetics*, 16(4):197–212, 2015.

[10] Dan L Nicolae, Eric Gamazon, Wei Zhang, Shiwei Duan, M Eileen Dolan, and Nancy J Cox. Trait-associated snps are more likely to be eqtls: annotation to enhance discovery from gwas. *PLoS genetics*, 6(4):e1000888, 2010.

[11] Margarita A Sazonova, Vasily V Sinyov, Anastasia I Ryzhkova, Marina D Sazonova, Tatiana V Kirichenko, Victoria A Khotina, Zukhra B Khasanova, Natalya A Doroschuk, Vasily P Karagodin, Alexander N Orekhov, et al. Some molecular and cellular stress mechanisms associated with neurodegenerative diseases and atherosclerosis. *International journal of molecular sciences*, 22(2):699, 2021.

[12] Ryan J Ramezani and Peter W Stacpoole. Sleep disorders associated with primary mitochondrial diseases. *Journal of Clinical Sleep Medicine*, 10(11):1233–1239, 2014.

[13] Catherine Slattery Slattery, Mark Agius, and Rashid Zaman. Bipolar disorder associated with paraneoplastic cerebellar degeneration: a case report. *Psychiatria Danubina*, 22(suppl 1.):137–138, 2010.

[14] Keri Martinowich, Robert J Schloesser, Husseini K Manji, et al. Bipolar disorder: from genes to behavior pathways. *The Journal of clinical investigation*, 119(4):726–736, 2009.

Table S1. Information of the eight GWAS summary database associated with eight psychiatric traits.

Psychiatric Trait	Number of variants	PMID	Ncase	Ncontrol	Ntotal
Alzheimer's Disease	12,688,339	34493870	90,338	1,036,225	1,126,563
Bipolar disorder	7,608,183	34002096	41,917	371,549	413,436
Anorexia nervosa	8,219,102	31308545	16,992	55,525	72,517
Depression	7,922,500	34586374	15,771	178,777	194,548
Insomnia	12,663,596	35835914	109,548	277,440	386,988
Intelligence	9,295,118	29942086	NA	NA	269,867
Parkinson's disease	12,222,969	31701892	112,612	2,474,079	2,586,691
Schizophrenia	7,659,768	35396580	76,755	243,649	320,404

Table S2. Effect size distribution estimation in eight psychiatric traits based on GWAS summary data. By means of GENESIS, the proportion of causal SNPs which possess non-zero effect size can be estimated. We adopt the M2 model in GENESIS which assumes that the effect size distribution of causal SNPs is a mixture of two normal distributions. The two normal distributions have zero means but unequal variances. The variances in the two causal SNP clusters and the heritability can be estimated by GENESIS as well.

Trait	Proportion of causal SNPs	Variance of cluster 1	Variance of cluster 2	heritability
Alzheimer's Disease	1.32×10^{-3}	6.86×10^{-6}	5.87×10^{-8}	0.00969
Bipolar disorder	1.24×10^{-2}	5.76×10^{-6}	3.38×10^{-7}	0.0763
Anorexia nervosa	1.03×10^{-2}	1.76×10^{-5}	9.84×10^{-7}	0.193
Depression	1.24×10^{-2}	3.66×10^{-6}	2.38×10^{-7}	0.0485
Insomnia	1.31×10^{-2}	1.75×10^{-6}	1.08×10^{-7}	0.0244
Intelligence	1.46×10^{-2}	1.31×10^{-5}	9.33×10^{-7}	0.205
Parkinson's disease	1.79×10^{-3}	7.13×10^{-6}	6.91×10^{-8}	0.0137
Schizophrenia	1.44×10^{-2}	2.75×10^{-6}	1.77×10^{-6}	0.423

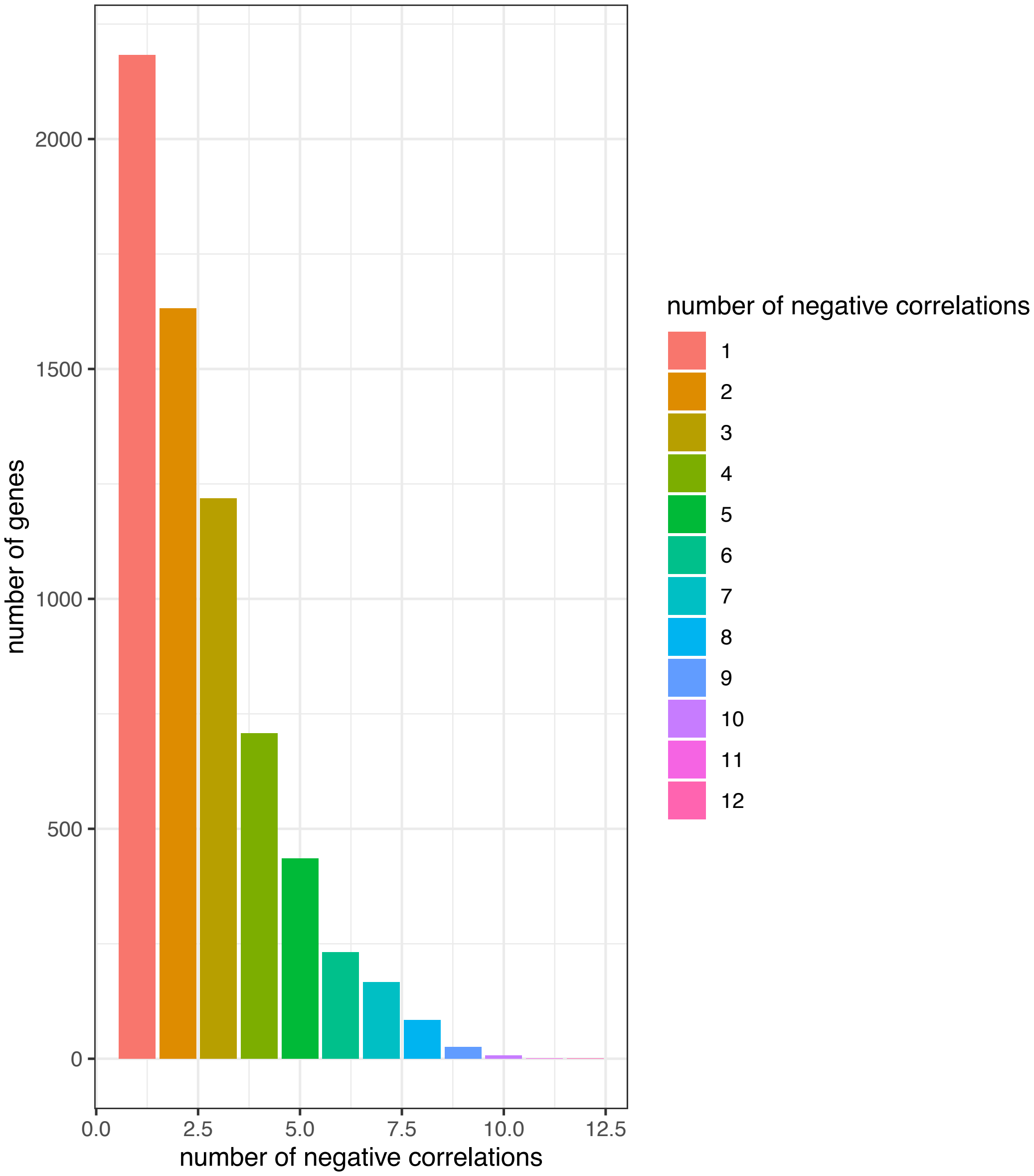

Fig. S1. Barplot for the number of genes possessing negative annotation correlations. For each of the analyzed 10940 genes, seven integrative annotations are incorporated into the imputation model, and the correlation between every pair of annotations in this gene is calculated. The x-axis represents the number of annotation pairs possessing negative correlations, the y-axis represents the number of genes possessing negative annotation correlations.

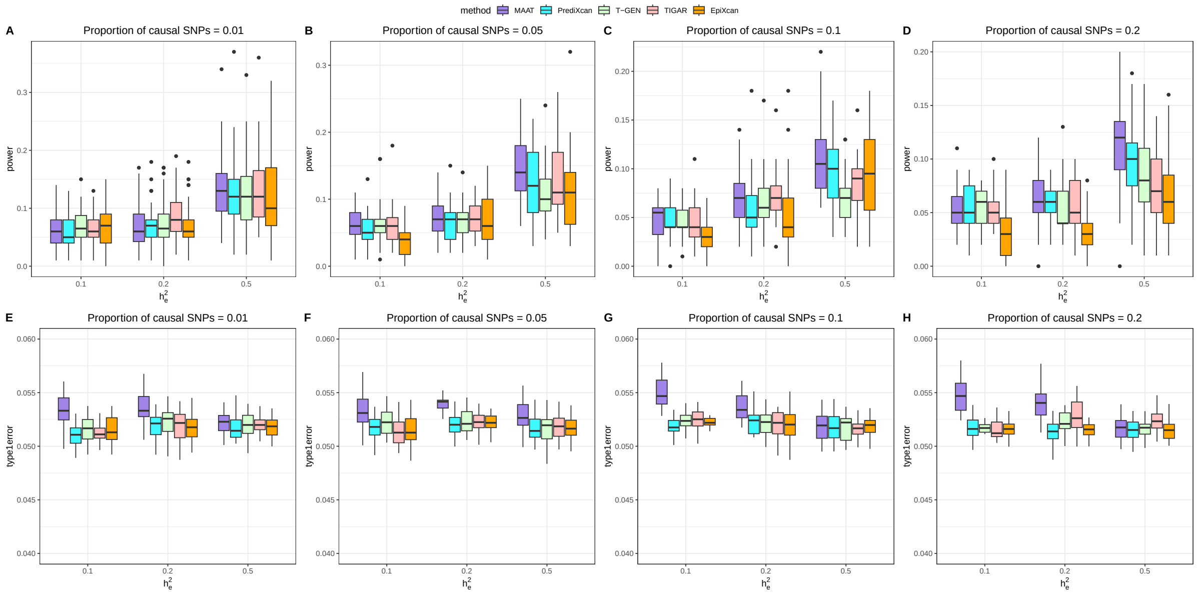

Fig. S2. Performance of power in simulation studies with phenotypic heritability h_p^2 equal to 0.1. We compare MAAT with PrediXcan, T-GEN, TIGAR and EpiXcan under different settings to evaluate the performance of association test power. The true proportion of causal SNPs p_{cs} is set to 0.01, 0.05, 0.1 and 0.2 respectively (A-D). The x-axis refers to the expression heritability h_e^2, which is set to 0.1, 0.2 and 0.5 respectively. At each replication of a (p_{cs}, h_e^2) setting, the phenotype is replicated for 100 times, the power is calculated as the proportion of p-values reaching the significant level among the 100 replications. The boxplots depict the distribution of power for each scenario and each method.

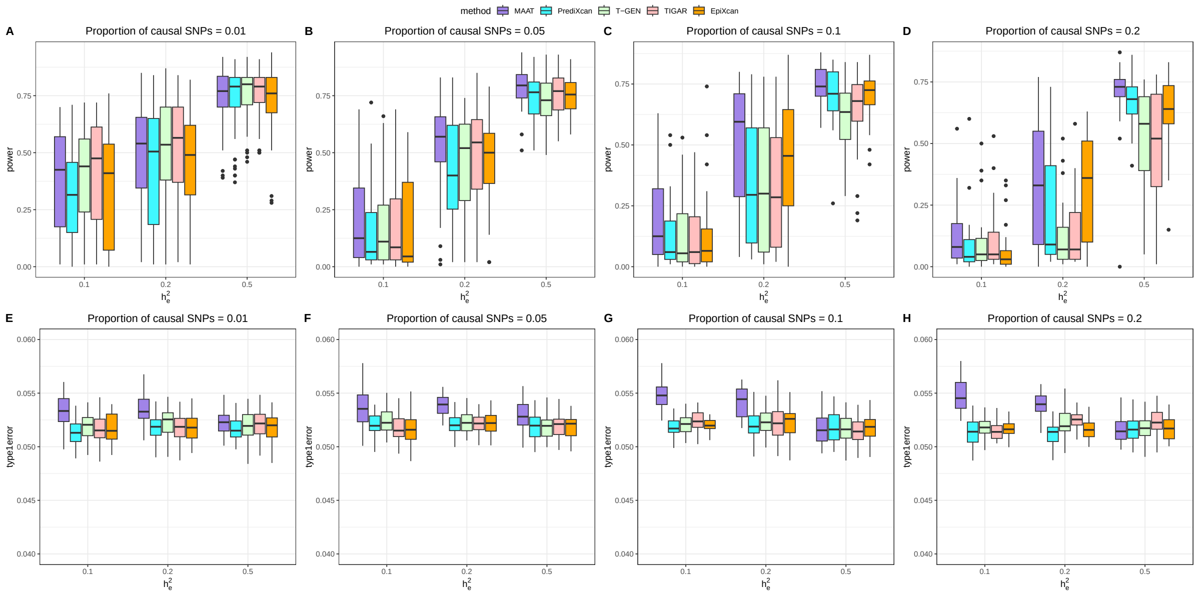

Fig. S3. Performance of power in simulation studies with phenotypic heritability h_p^2 equal to 0.5. We compare MAAT with PrediXcan, T-GEN, TIGAR and EpiXcan under different settings to evaluate the performance of association test power. The true proportion of causal SNPs p_{cs} is set to 0.01, 0.05, 0.1 and 0.2 respectively (A-D). The x-axis refers to the expression heritability h_e^2, which is set to 0.1, 0.2 and 0.5 respectively. At each replication of a (p_{cs}, h_e^2) setting, the phenotype is replicated for 100 times, the power is calculated as the proportion of p-values reaching the significant level among the 100 replications. The boxplots depict the distribution of power for each scenario and each method.

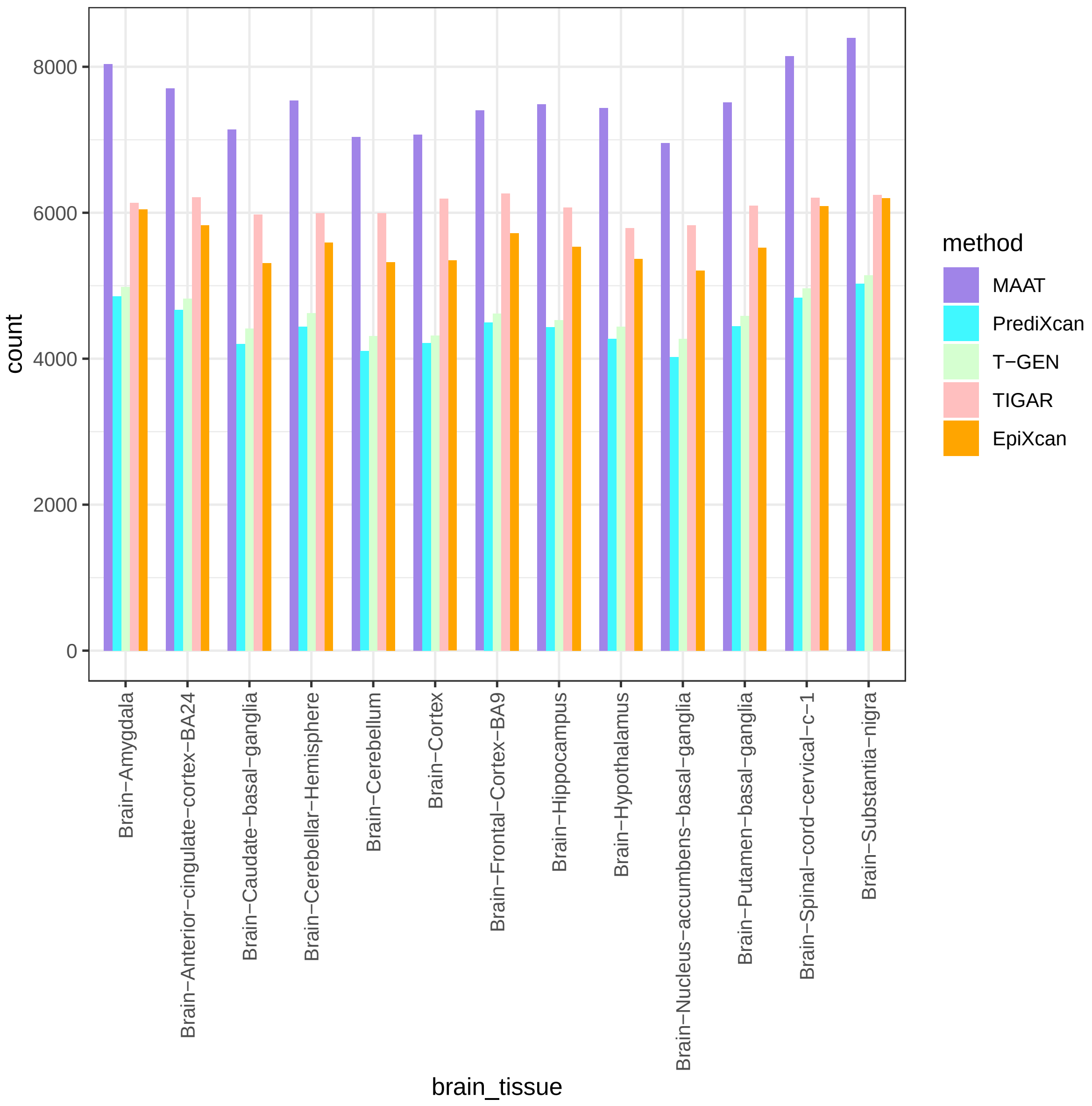

Fig. S4. Test R^2 in GTEx V8 database across 13 brain tissues for five methods. After gene expression imputation in the ROS/MAP reference panel, external validation is implemented in the independent GTEx V8 database. The x-axis refers to 13 different brain tissues, the y-axis refers to the number of genes with test R^2 greater than 0.005 for five different methods.

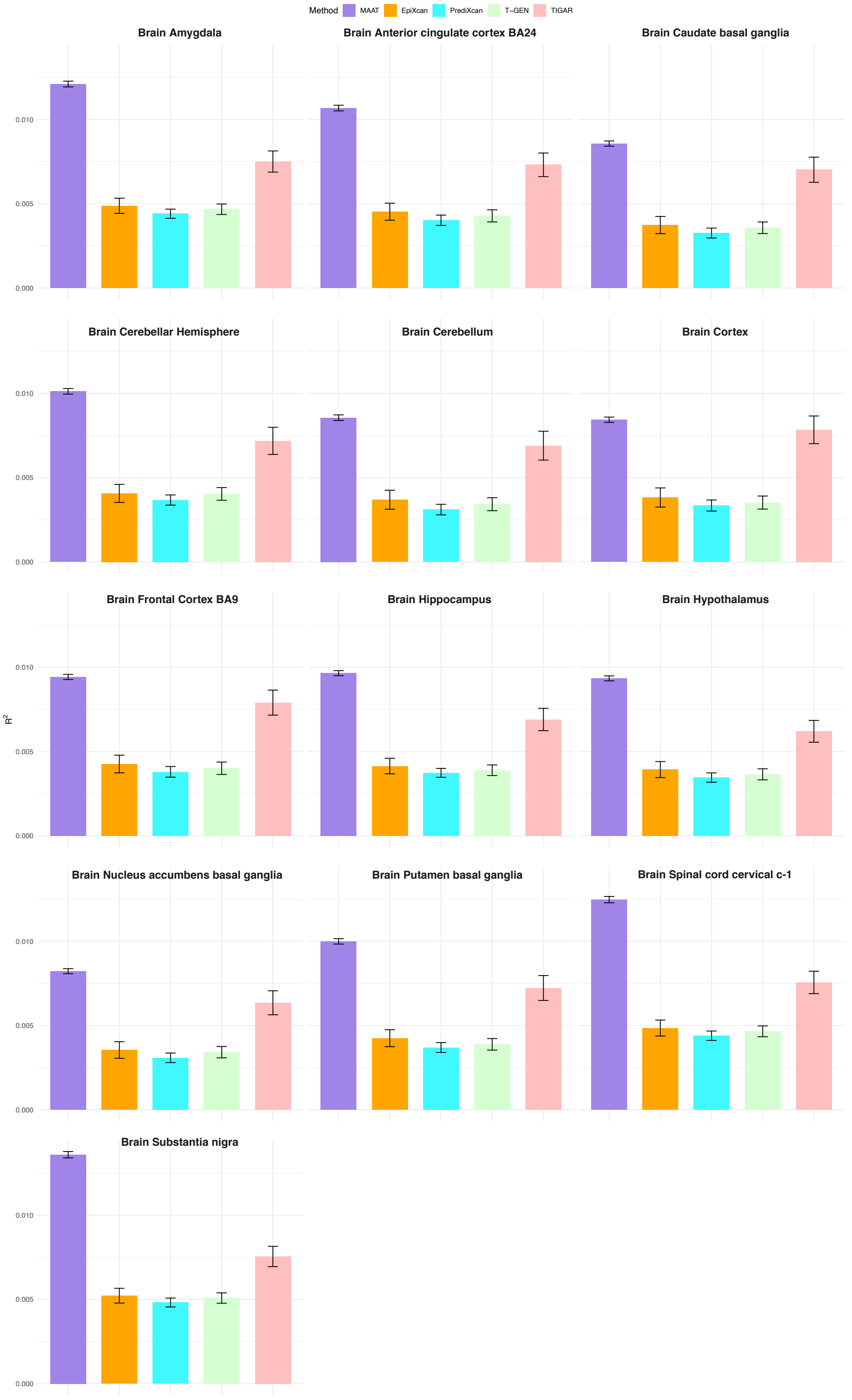

Fig. S5. Distribution of test R^2 in GTEx V8 database across 13 brain tissues for five methods. After gene expression imputation in the ROS/MAP reference panel, external validation is implemented in 13 different brain tissues in the independent GTEx V8 database. Each subfigure represents the distribution of validation R^2 for five methods on a specific brain tissue. The height of the bar indicates the median value of the validation R^2, the length of the error bar represents the standard deviation of R^2.

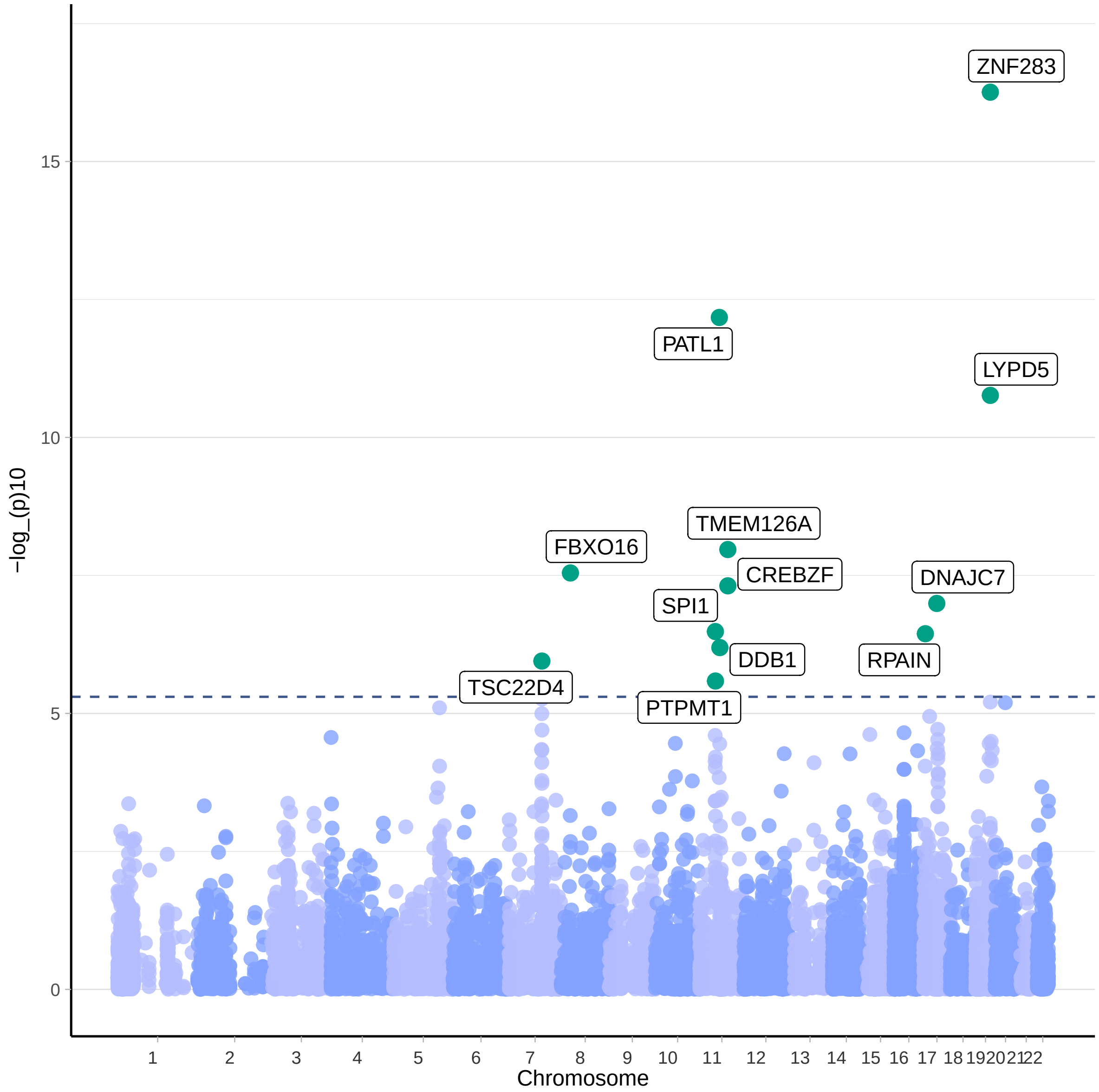

Fig. S6. TWAS Manhattan plot in Alzheimer's disease. Blue dashed horizontal line marks the genome-wide significance threshold at 5×10^{-6} (Bonferroni threshold corrected for 10,000 genes). Significant genes are indicated with green circles, and their names are labeled.

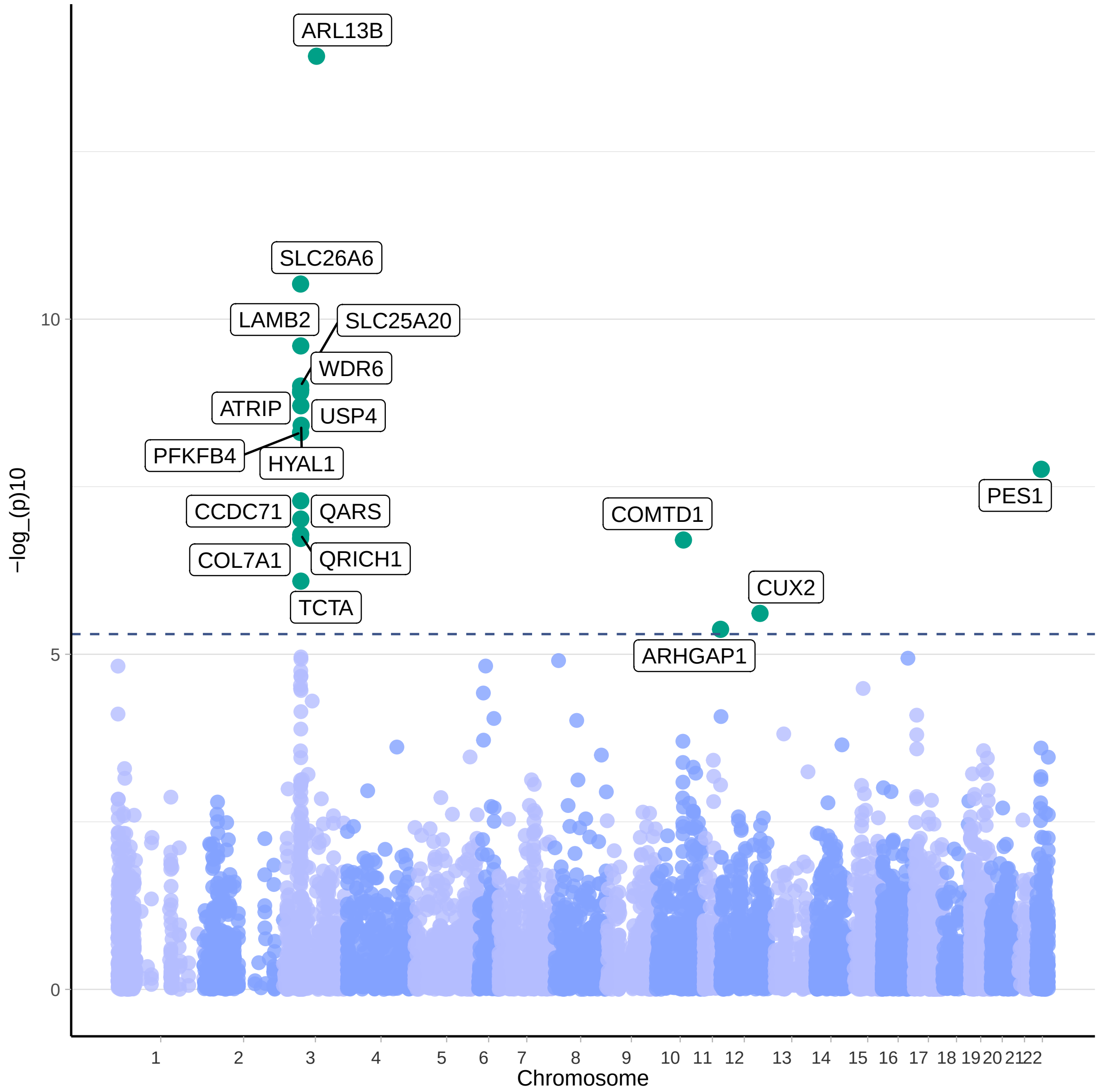

Fig. S7. TWAS Manhattan plot in anorexia nervosa. Blue dashed horizontal line marks the genome-wide significance threshold at 5×10^{-6} (Bonferroni threshold corrected for 10,000 genes). Significant genes are indicated with green circles, and their names are labeled.

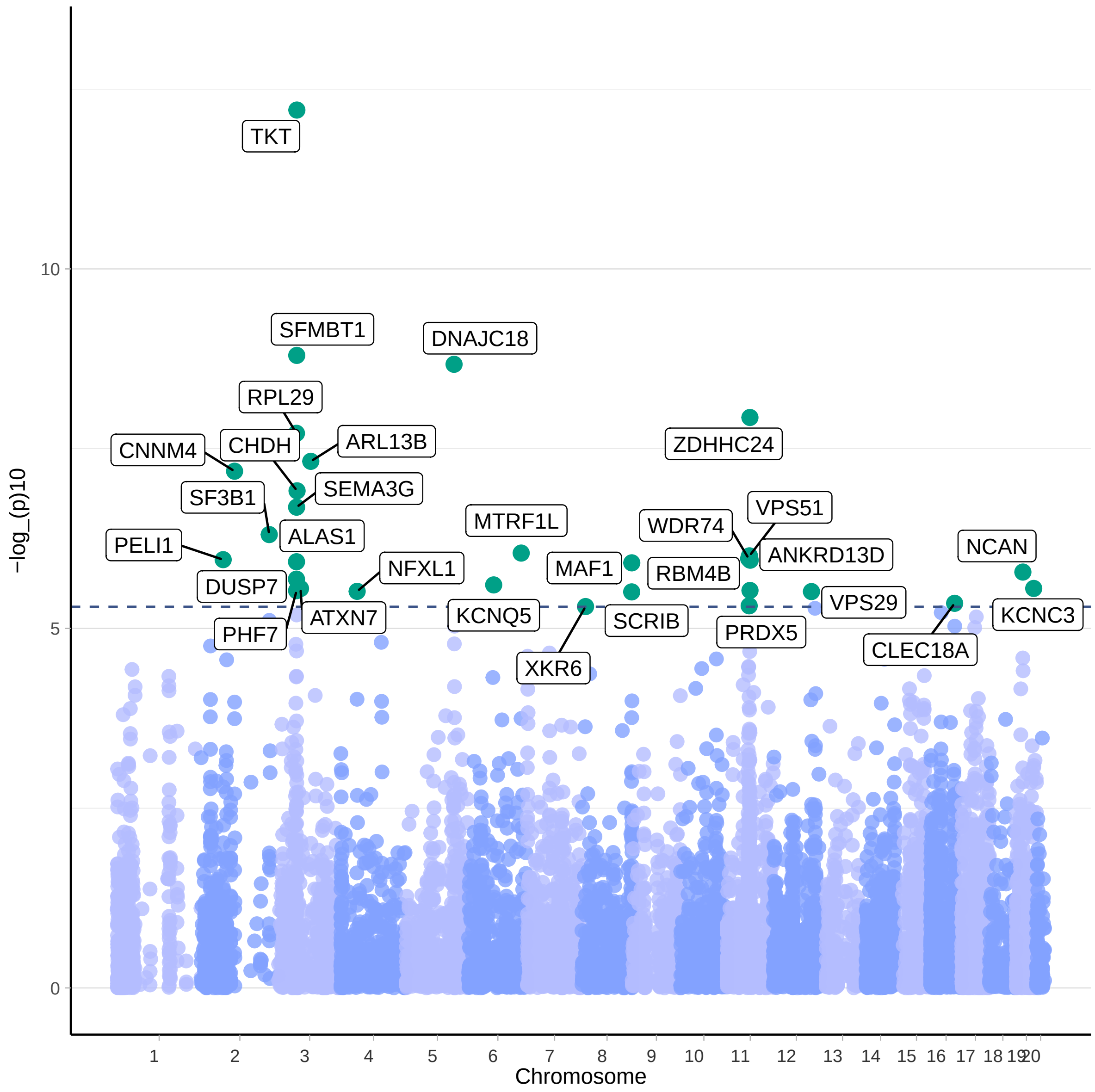

Fig. S8. TWAS Manhattan plot in bipolar disorder. Blue dashed horizontal line marks the genome-wide significance threshold at 5×10^{-6} (Bonferroni threshold corrected for 10,000 genes). Significant genes are indicated with green circles, and their names are labeled.

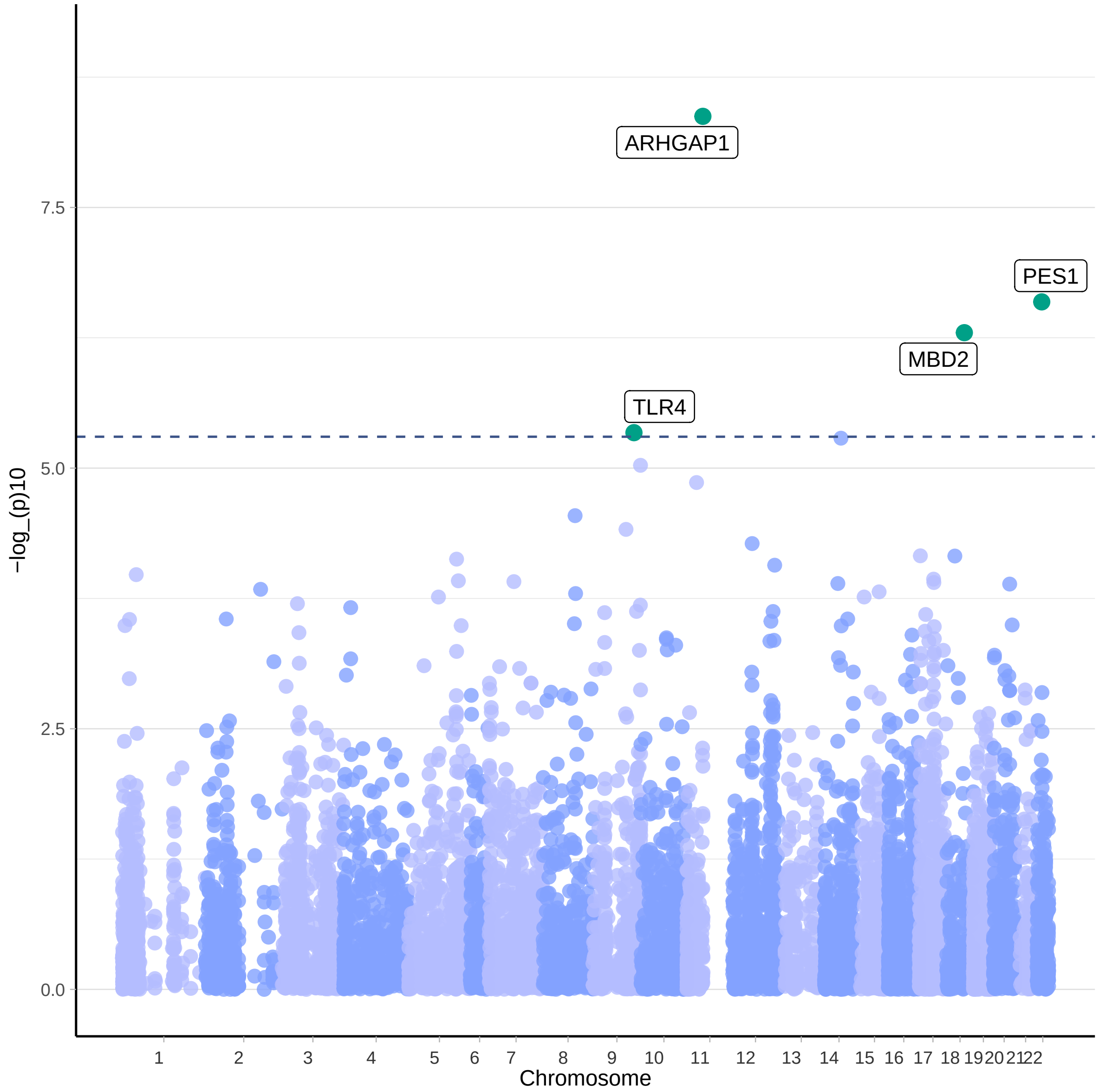

Fig. S9. TWAS Manhattan plot in depression. Blue dashed horizontal line marks the genome-wide significance threshold at 5×10^{-6} (Bonferroni threshold corrected for 10,000 genes). Significant genes are indicated with green circles, and their names are labeled.

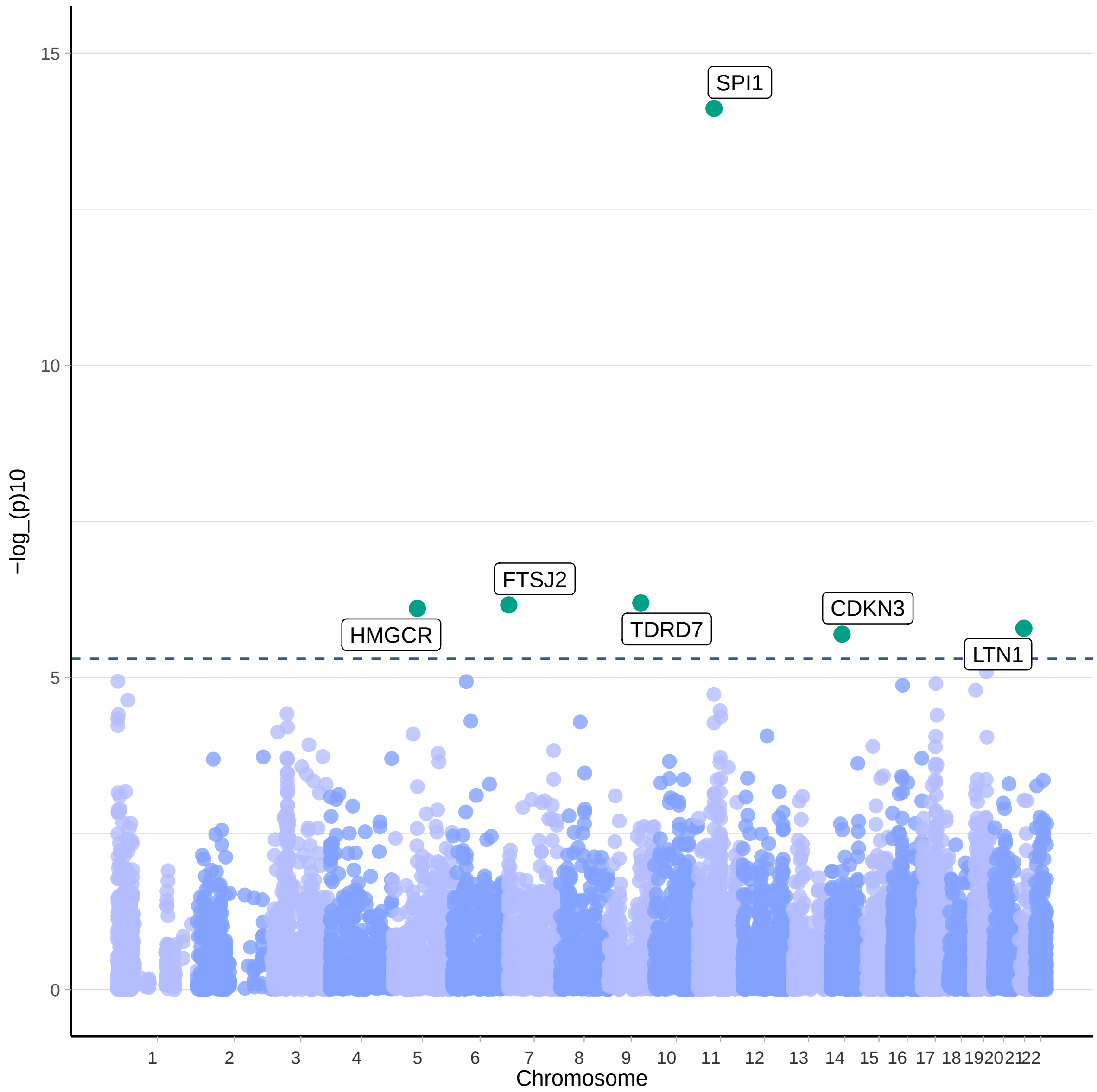

Fig. S10. TWAS Manhattan plot in insomnia. Blue dashed horizontal line marks the genome-wide significance threshold at 5×10^{-6} (Bonferroni threshold corrected for 10,000 genes). Significant genes are indicated with green circles, and their names are labeled.

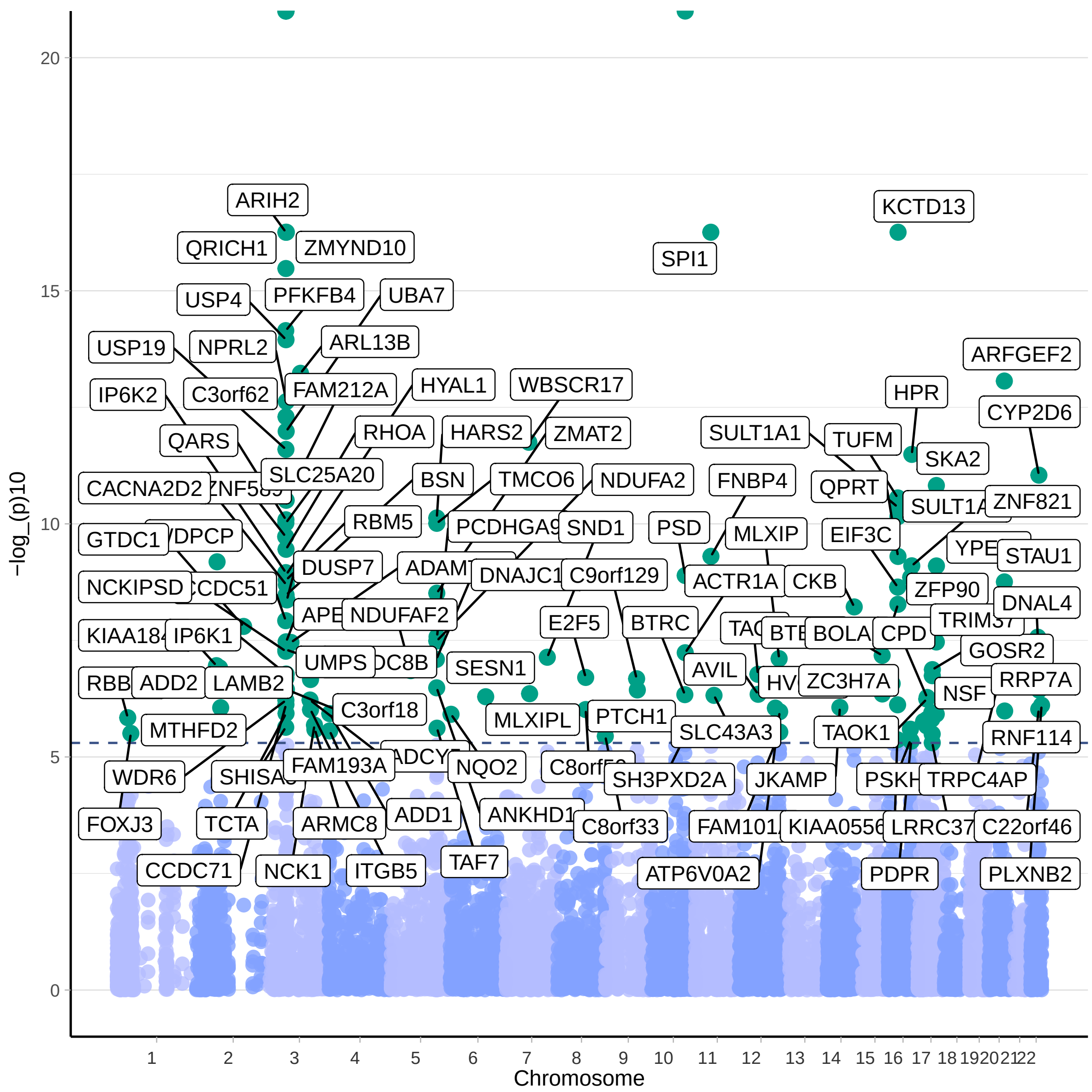

Fig. S11. TWAS Manhattan plot in intelligence. Blue dashed horizontal line marks the genome-wide significance threshold at 5×10^{-6} (Bonferroni threshold corrected for 10,000 genes). Significant genes are indicated with green circles, and their names are labeled.

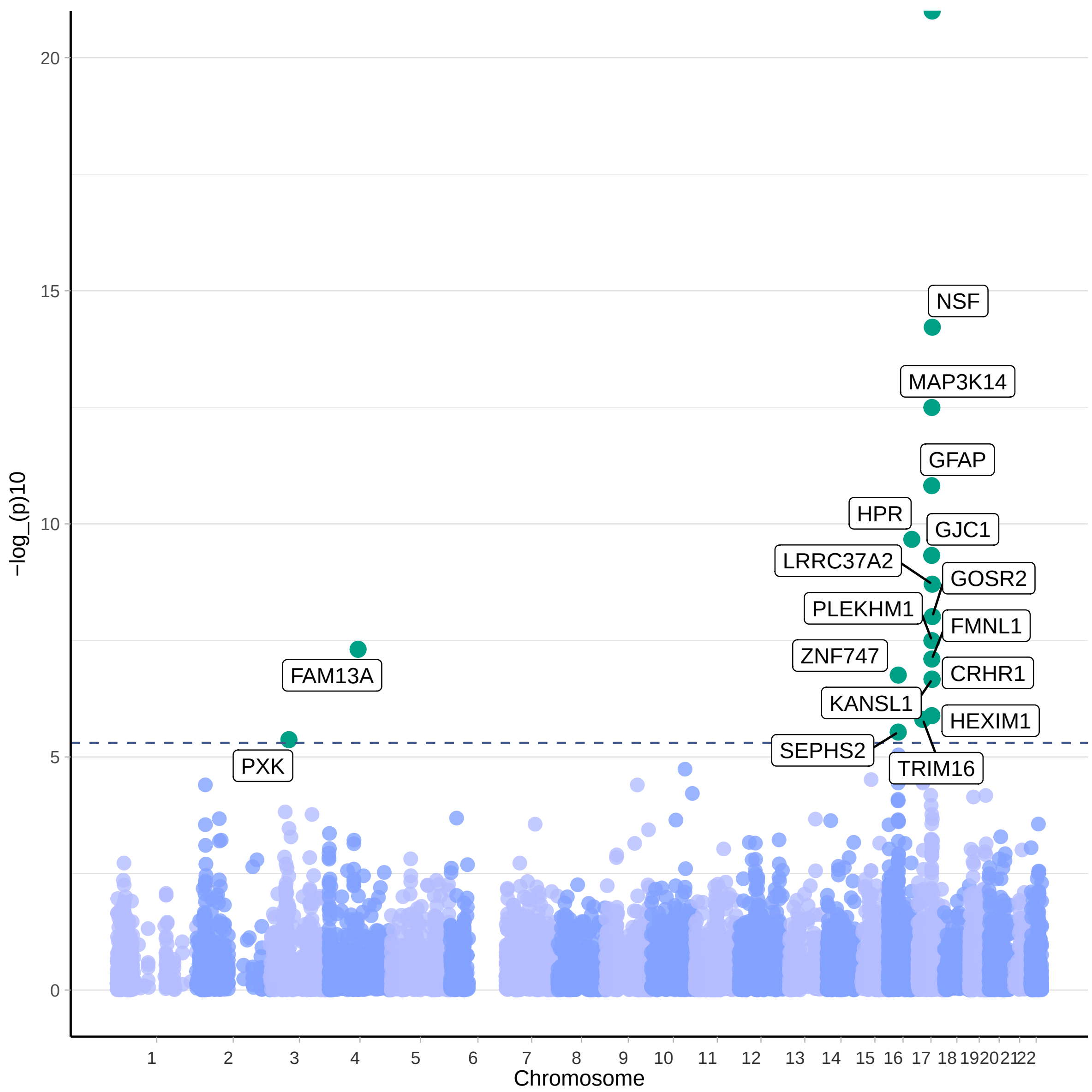

Fig. S12. TWAS Manhattan plot in Parkinson's disease. Blue dashed horizontal line marks the genome-wide significance threshold at 5×10^{-6} (Bonferroni threshold corrected for 10,000 genes). Significant genes are indicated with green circles, and their names are labeled.

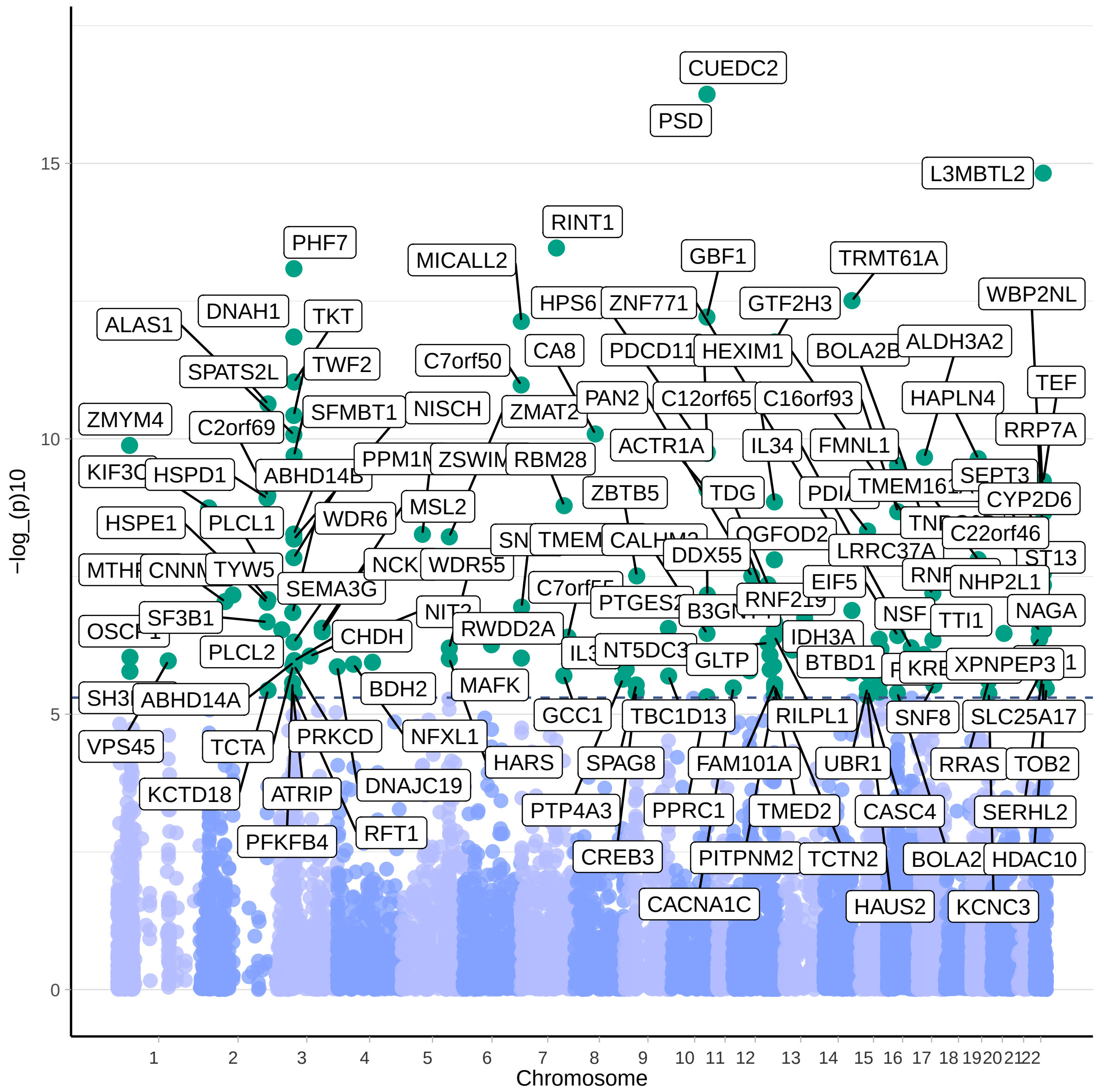

Fig. S13. TWAS Manhattan plot in schizophrenia. Blue dashed horizontal line marks the genome-wide significance threshold at 5×10^{-6} (Bonferroni threshold corrected for 10,000 genes). Significant genes are indicated with green circles, and their names are labeled.

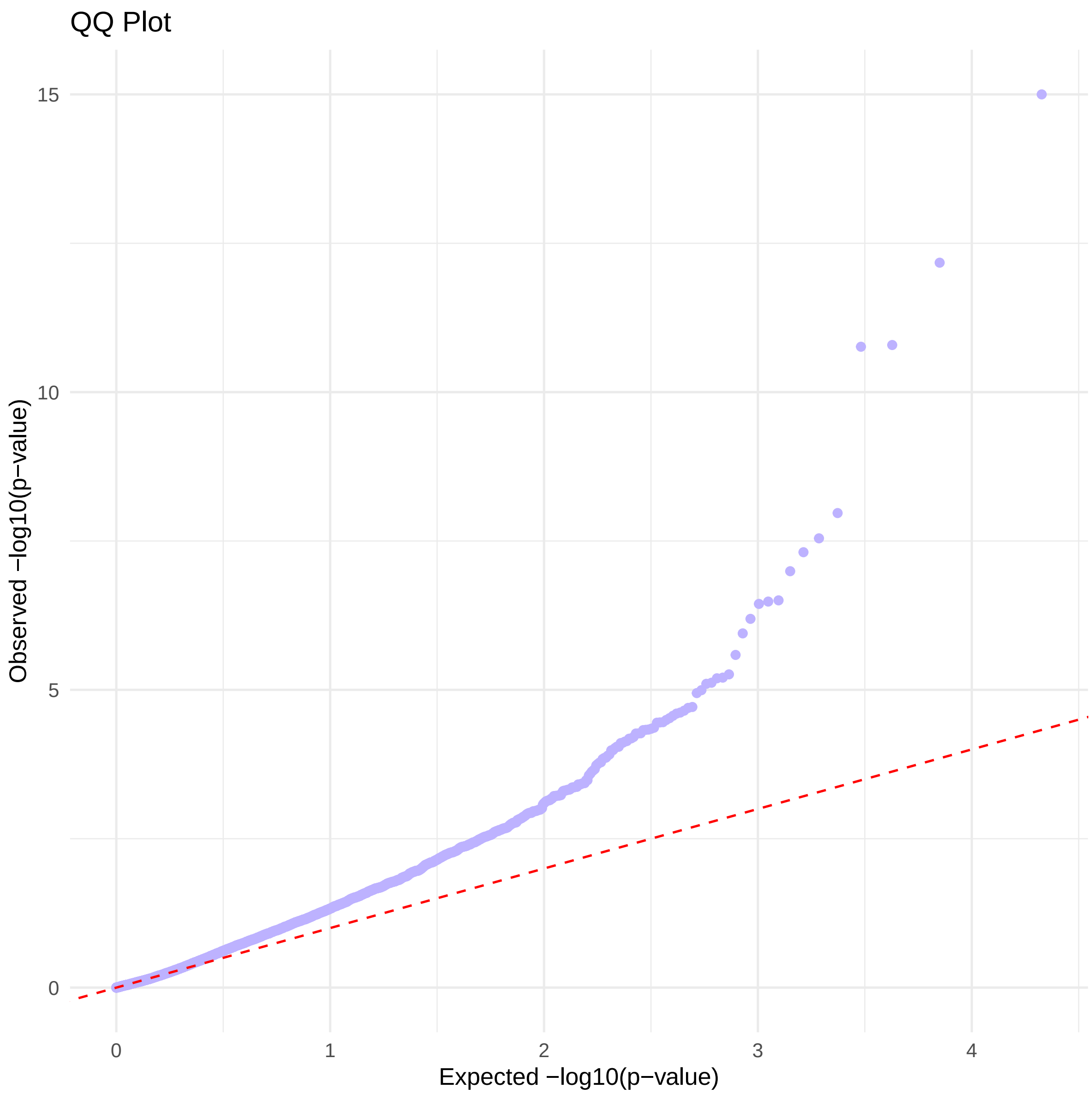

Fig. S14. QQ plot for the MAAT method in Alzheimer's disease. Each dot respresents a gene, the y-axis represents the observed $-\log 10$ (p-value), x-axis represents the expected $-\log 10$ (p-value). For p-values less than 10^{-15}, we truncated them to 10^{-15} for presentation.

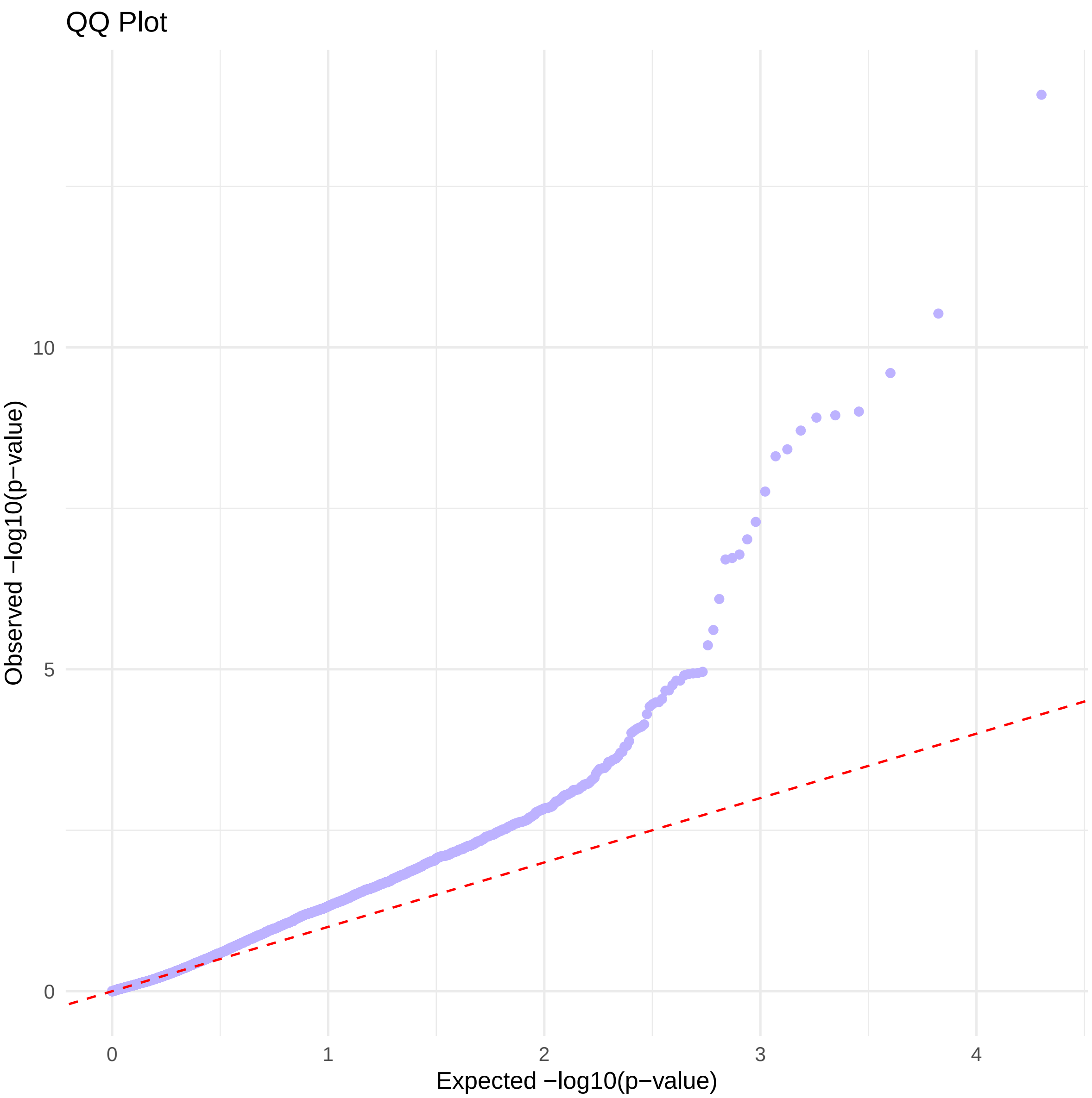

Fig. S15. QQ plot for the MAAT method in anorexia nervosa. Each dot respresents a gene, the y-axis represents the observed $-\log 10$ (p-value), x-axis represents the expected $-\log 10$ (p-value). For p-values less than 10^{-15}, we truncated them to 10^{-15} for presentation.

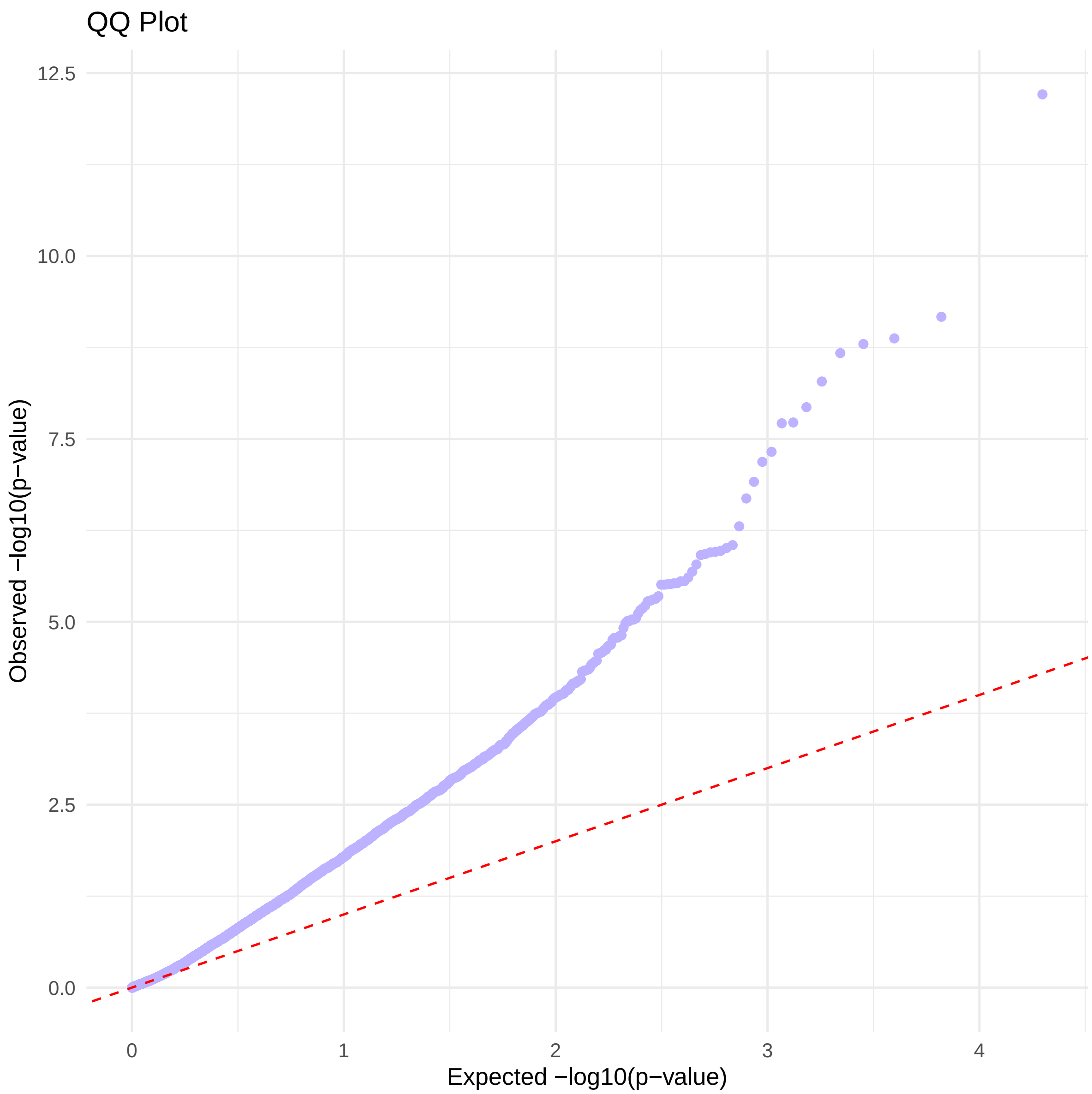

Fig. S16. QQ plot for the MAAT method in bipolar disorder. Each dot respresents a gene, the y-axis represents the observed $-\log 10$ (p-value), x-axis represents the expected $-\log 10$ (p-value). For p-values less than 10^{-15}, we truncated them to 10^{-15} for presentation.

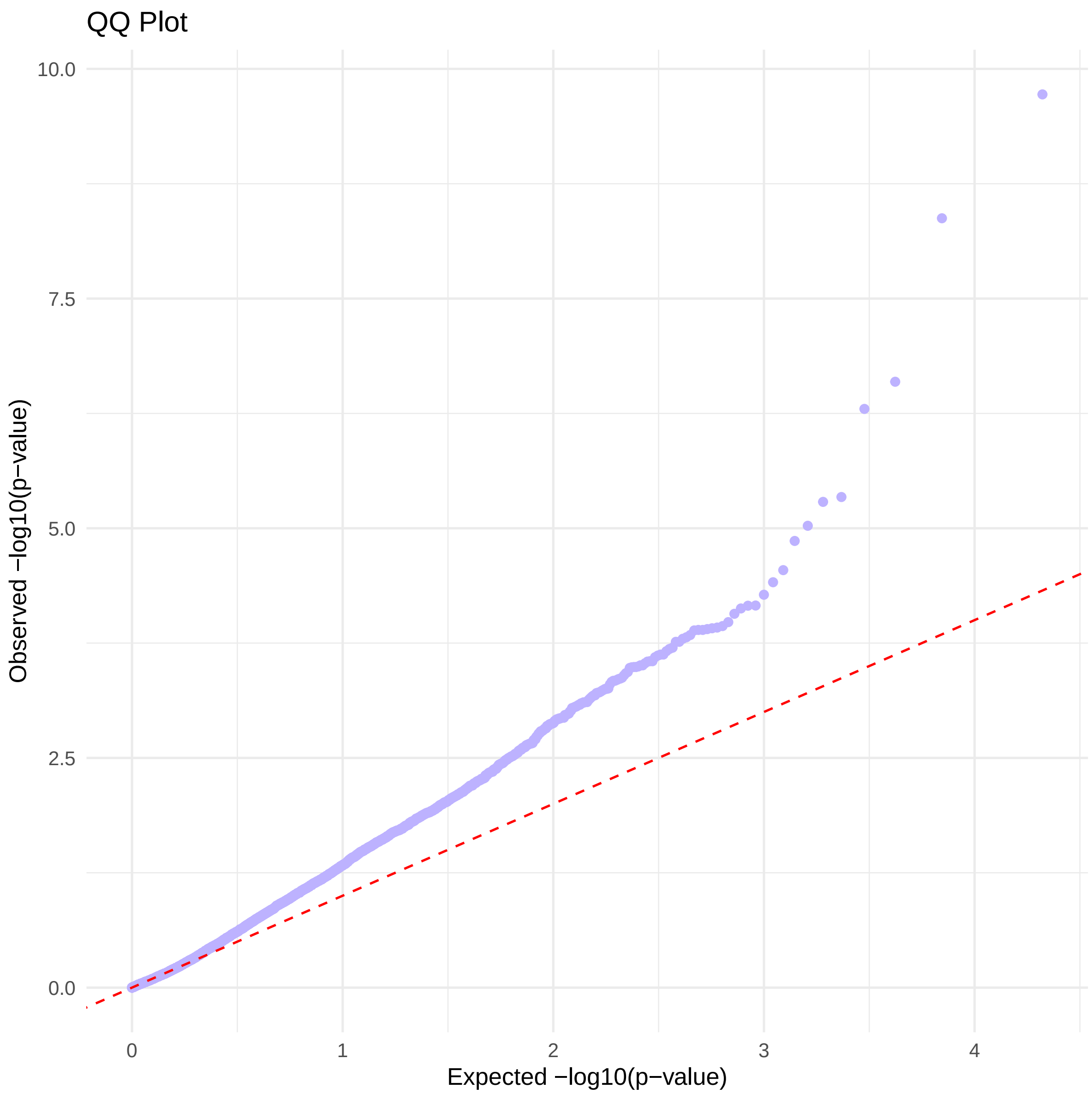

Fig. S17. QQ plot for the MAAT method in depression. Each dot respresents a gene, the y-axis represents the observed $-\log 10$ (p-value), x-axis represents the expected $-\log 10$ (p-value). For p-values less than 10^{-15}, we truncated them to 10^{-15} for presentation.

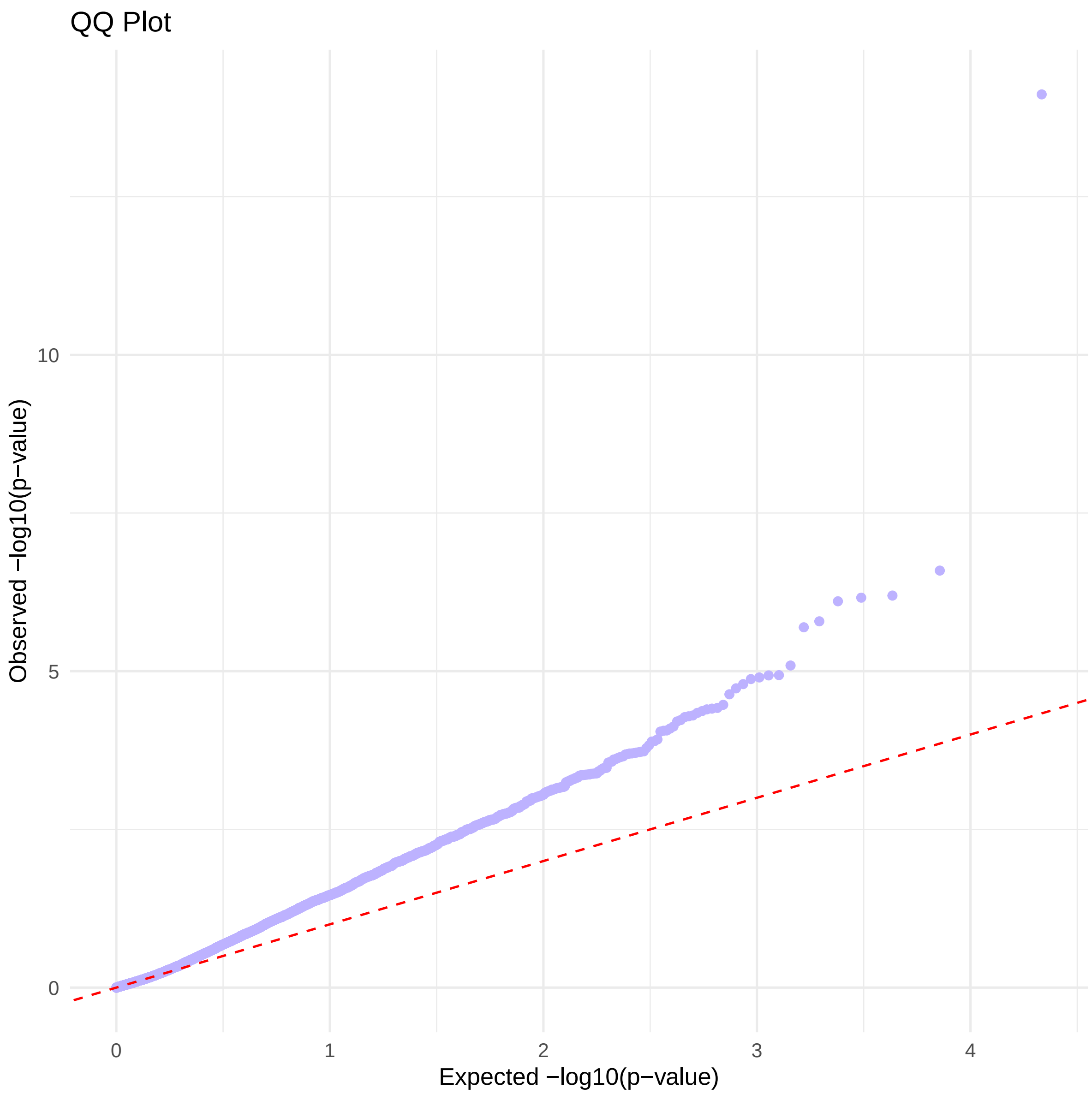

Fig. S18. QQ plot for the MAAT method in insomnia. Each dot respresents a gene, the y-axis represents the observed $-\log 10$ (p-value), x-axis represents the expected $-\log 10$ (p-value). For p-values less than 10^{-15}, we truncated them to 10^{-15} for presentation.

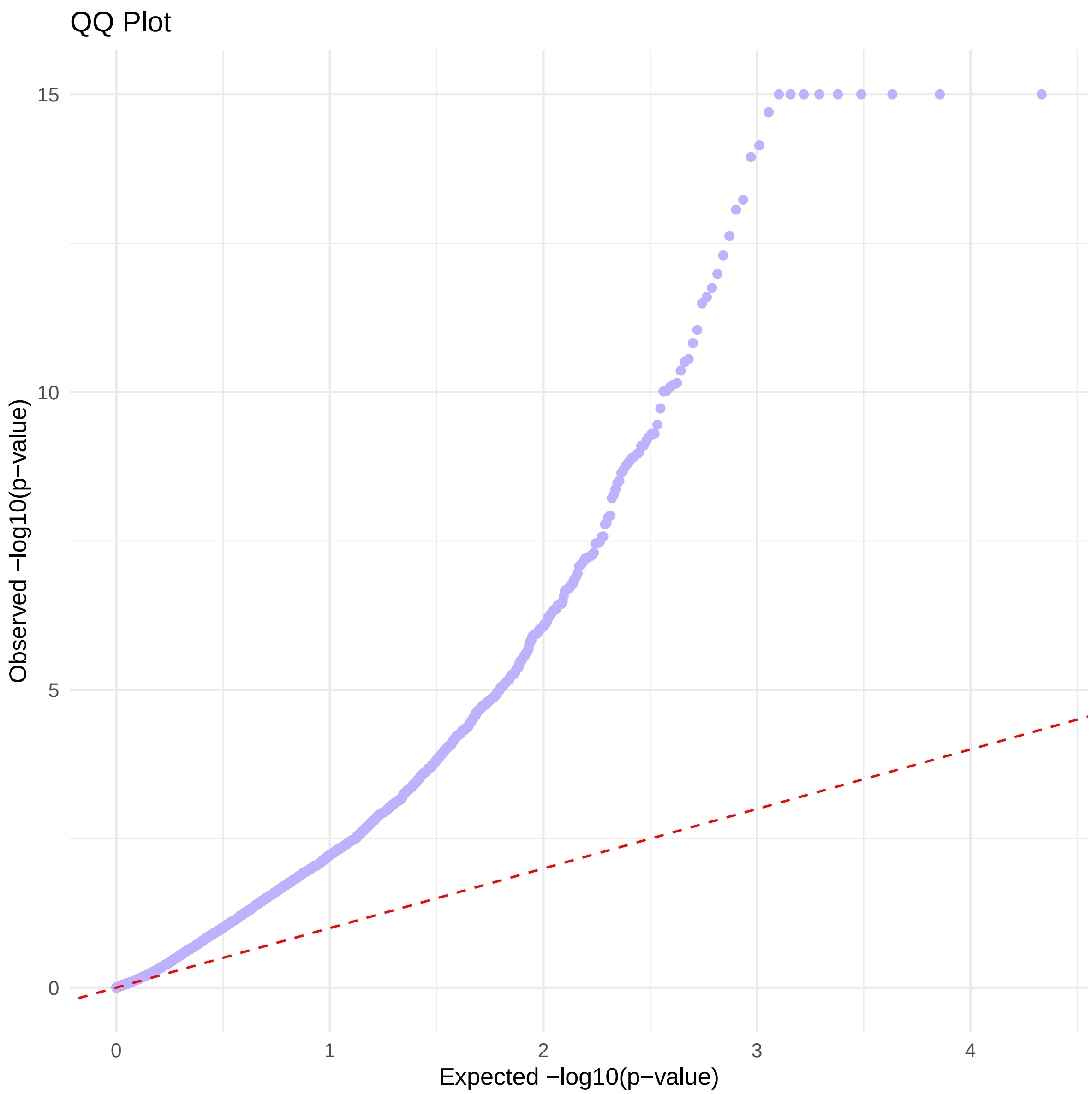

Fig. S19. QQ plot for the MAAT method in intelligence. Each dot respresents a gene, the y-axis represents the observed $-\log 10$ (p-value), x-axis represents the expected $-\log 10$ (p-value). For p-values less than 10^{-15}, we truncated them to 10^{-15} for presentation.

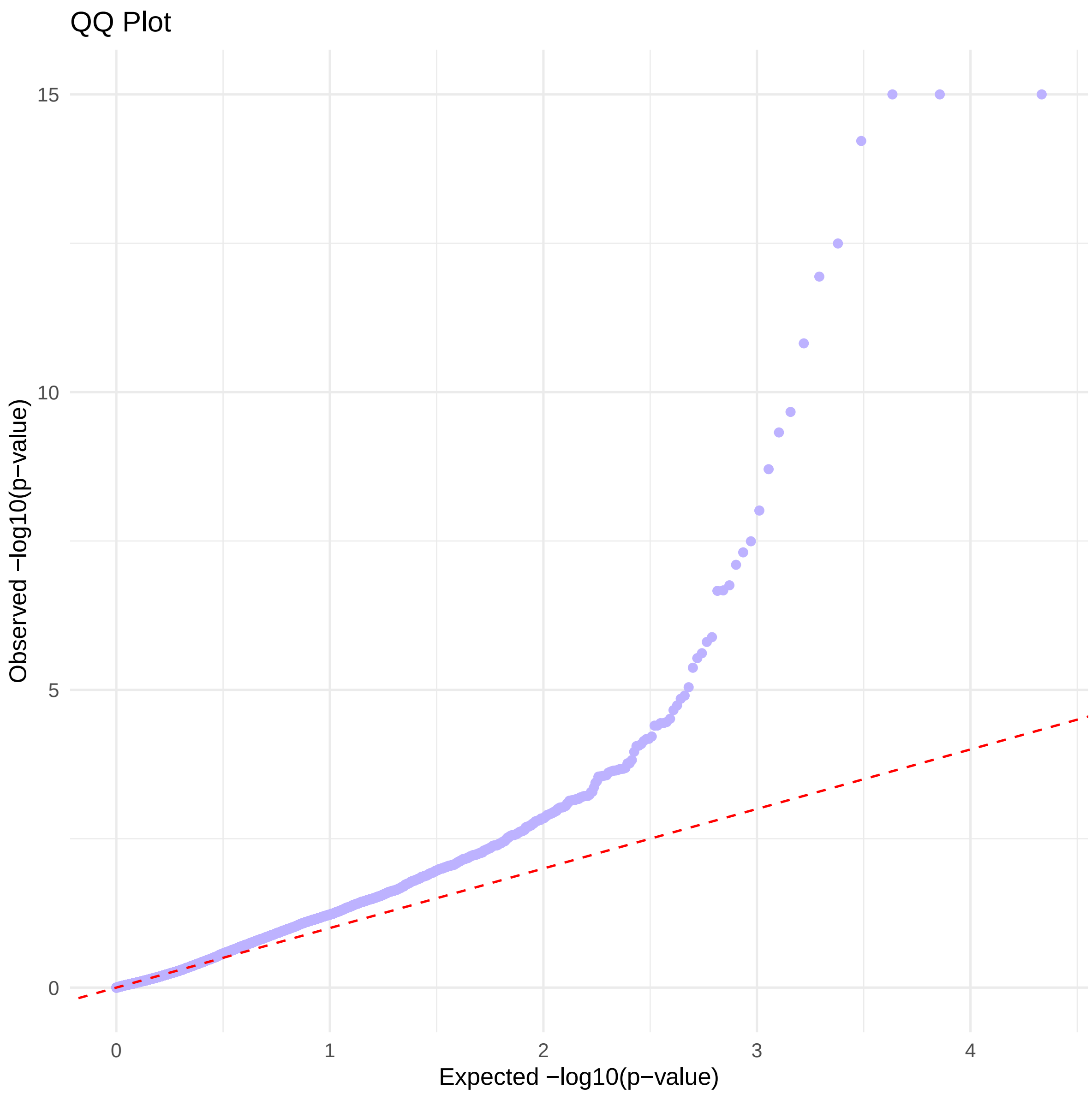

Fig. S20. QQ plot for the MAAT method in Parkinson's disease. Each dot respresents a gene, the y-axis represents the observed $-\log 10$ (p-value), x-axis represents the expected $-\log 10$ (p-value). For p-values less than 10^{-15}, we truncated them to 10^{-15} for presentation.

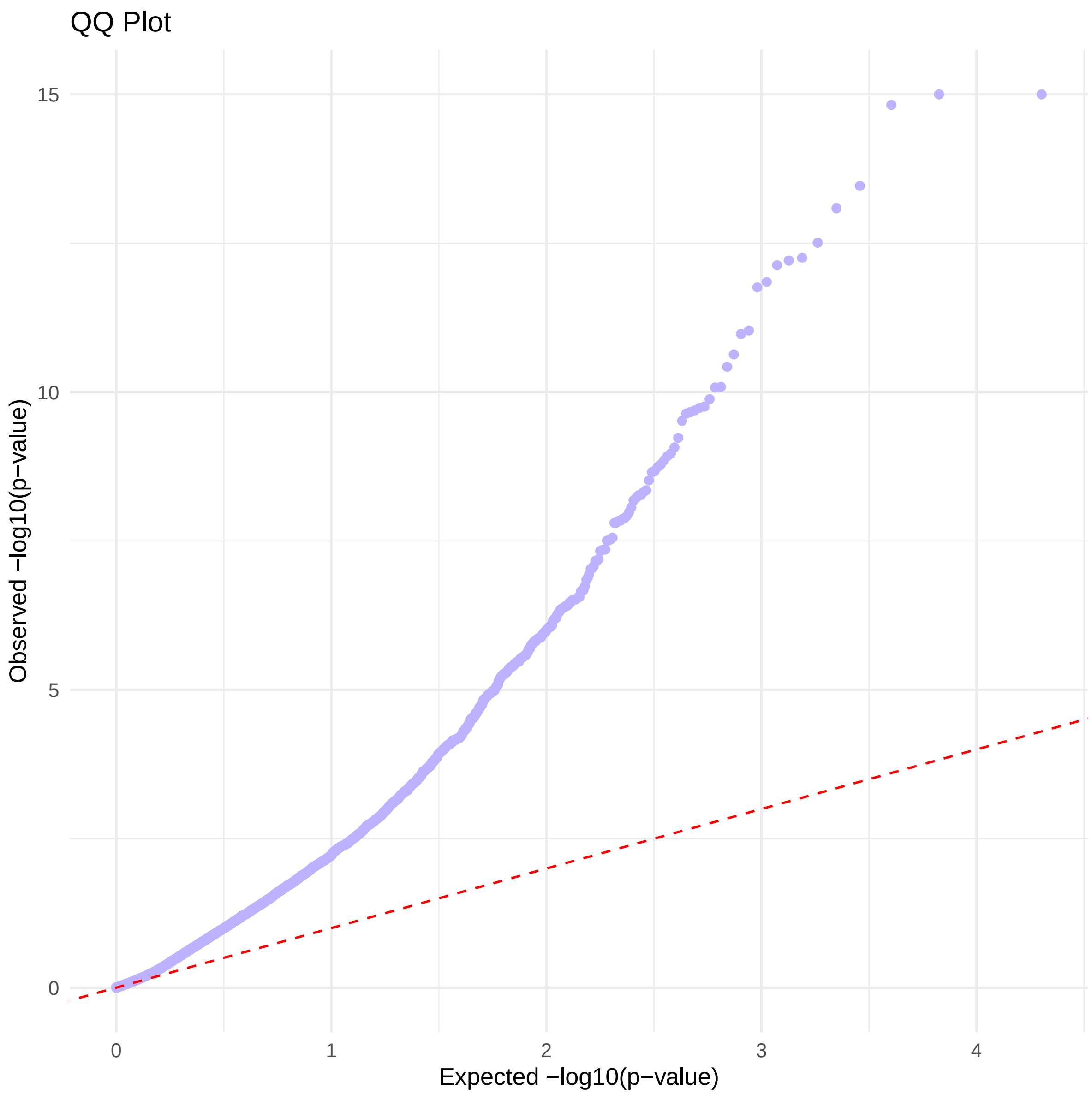

Fig. S21. QQ plot for the MAAT method in schizophrenia. Each dot respresents a gene, the y-axis represents the observed $-\log 10$ (p-value), x-axis represents the expected $-\log 10$ (p-value). For p-values less than 10^{-15}, we truncated them to 10^{-15} for presentation.

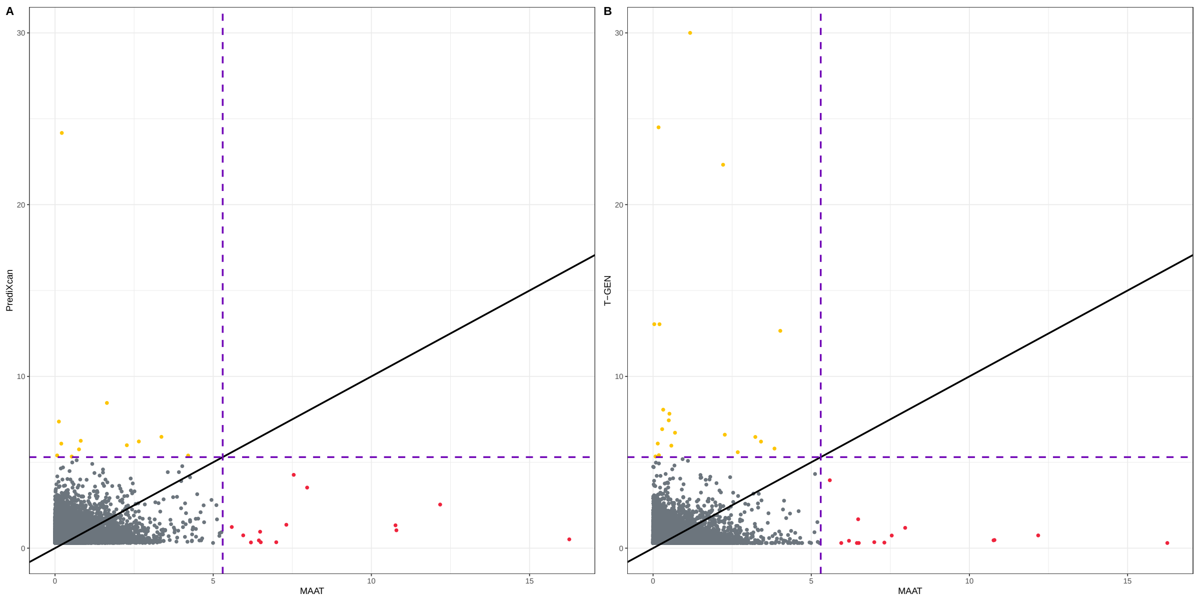

Fig. S22. Scatterplot of p-values for gene-trait associations comparing MAAT with PrediXcan (left) and T-GEN (right) in Alzheimer's disease GWAS data. Each dot represents a gene. The x-axis refers to the $-\log 10$ (p-value) indicating the TWAS significance level calculated by MAAT, and the y-axis refers to the $-\log 10$ (p-value) indicating the TWAS significance level calculated by PrediXcan (left) or T-GEN (right). The purple dashed line corresponds to the p-value cutoff of $5e-6$. Genes not reaching the significance level in both methods are represented with gray dots, genes reaching the significance level in MAAT while not reaching the significance level in other methods are represented with red dots, genes reaching the significance level in both methods are represented with cyan dots.

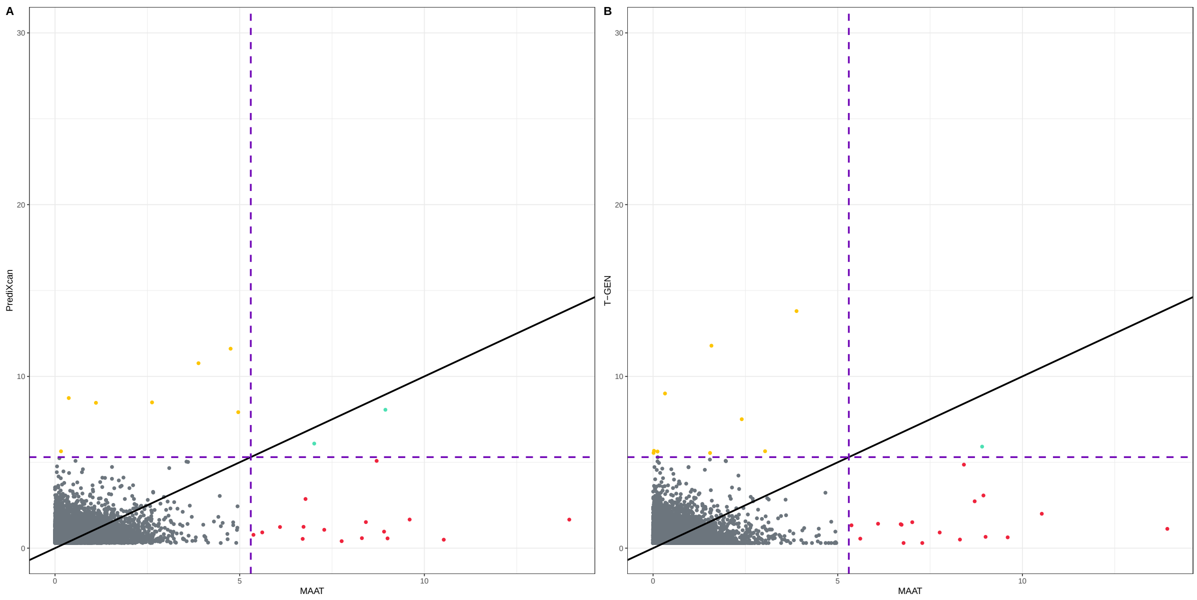

Fig. S23. Scatterplot of p-values for gene-trait associations comparing MAAT with PrediXcan (left) and T-GEN (right) in anorexia nervosa GWAS data. Each dot represents a gene. The x-axis refers to the $-\log 10$ (p-value) indicating the TWAS significance level calculated by MAAT, and the y-axis refers to the $-\log 10$ (p-value) indicating the TWAS significance level calculated by PrediXcan (left) or T-GEN (right). The purple dashed line corresponds to the p-value cutoff of $5e-6$. Genes not reaching the significance level in both methods are represented with gray dots, genes reaching the significance level in MAAT while not reaching the significance level in other methods are represented with red dots, genes reaching the significance level in both methods are represented with cyan dots.

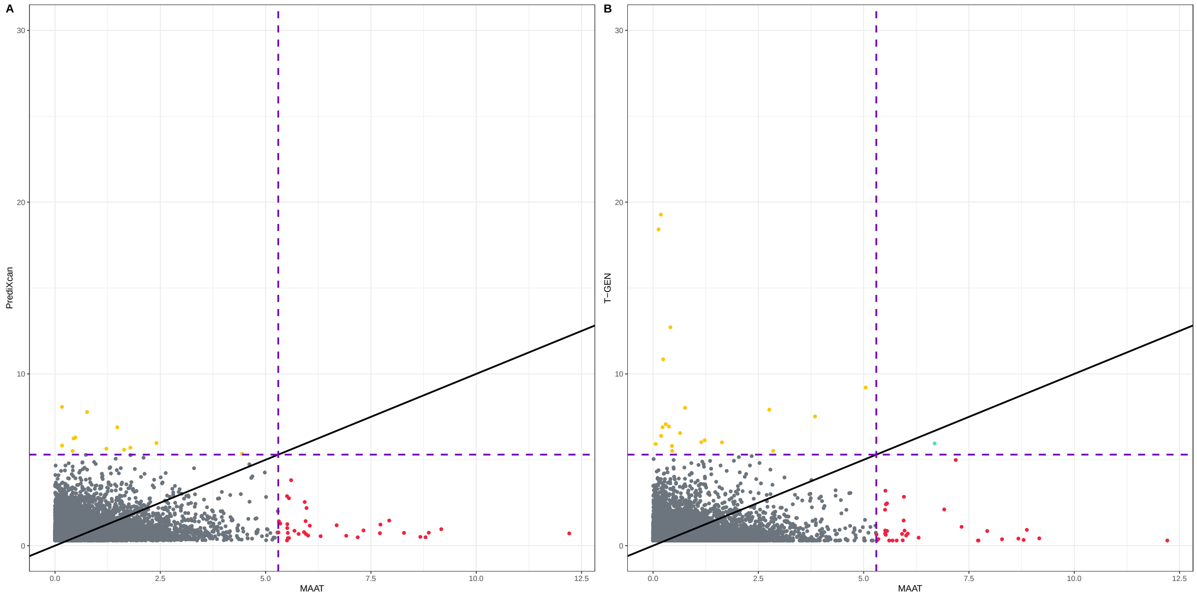

Fig. S24. Scatterplot of p-values for gene-trait associations comparing MAAT with PrediXcan (left) and T-GEN (right) in bipolar disorder GWAS data. Each dot represents a gene. The x-axis refers to the $-\log 10$ (p-value) indicating the TWAS significance level calculated by MAAT, and the y-axis refers to the $-\log 10$ (p-value) indicating the TWAS significance level calculated by PrediXcan (left) or T-GEN (right). The purple dashed line corresponds to the p-value cutoff of $5e-6$. Genes not reaching the significance level in both methods are represented with gray dots, genes reaching the significance level in MAAT while not reaching the significance level in other methods are represented with red dots, genes reaching the significance level in both methods are represented with cyan dots.

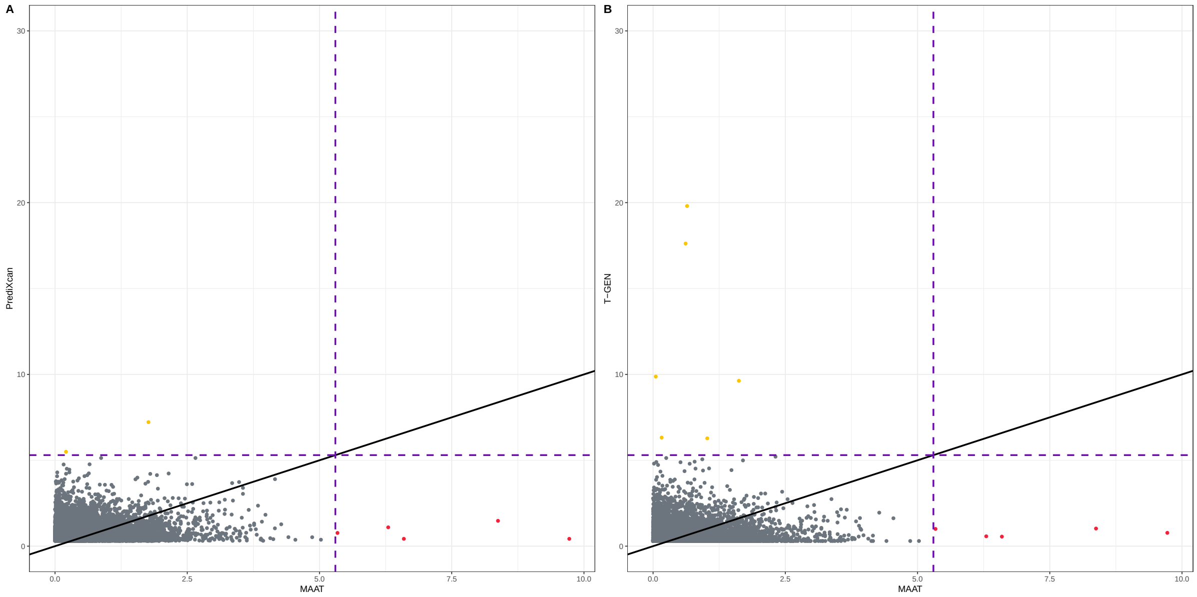

Fig. S25. Scatterplot of p-values for gene-trait associations comparing MAAT with PrediXcan (left) and T-GEN (right) in depression GWAS data. Each dot represents a gene. The x-axis refers to the $-\log 10$ (p-value) indicating the TWAS significance level calculated by MAAT, and the y-axis refers to the $-\log 10$ (p-value) indicating the TWAS significance level calculated by PrediXcan (left) or T-GEN (right). The purple dashed line corresponds to the p-value cutoff of $5e-6$. Genes not reaching the significance level in both methods are represented with gray dots, genes reaching the significance level in MAAT while not reaching the significance level in other methods are represented with red dots, genes reaching the significance level in both methods are represented with cyan dots.

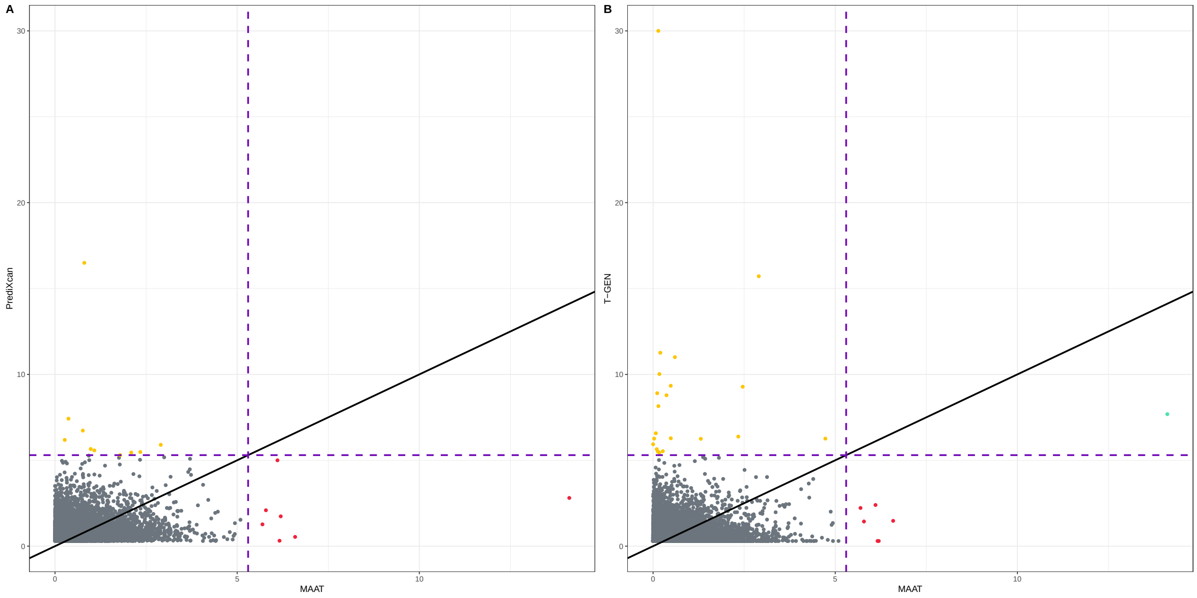

Fig. S26. Scatterplot of p-values for gene-trait associations comparing MAAT with PrediXcan (left) and T-GEN (right) in insomnia GWAS data. Each dot represents a gene. The x-axis refers to the $-\log 10$ (p-value) indicating the TWAS significance level calculated by MAAT, and the y-axis refers to the $-\log 10$ (p-value) indicating the TWAS significance level calculated by PrediXcan (left) or T-GEN (right). The purple dashed line corresponds to the p-value cutoff of $5e-6$. Genes not reaching the significance level in both methods are represented with gray dots, genes reaching the significance level in MAAT while not reaching the significance level in other methods are represented with red dots, genes reaching the significance level in both methods are represented with cyan dots.

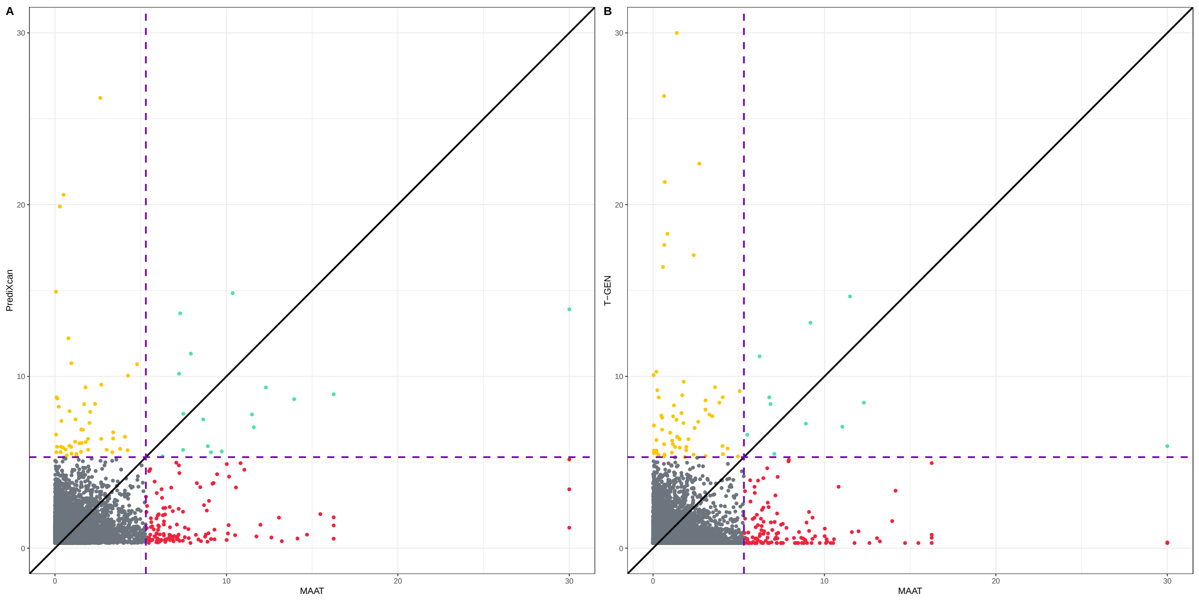

Fig. S27. Scatterplot of p-values for gene-trait associations comparing MAAT with PrediXcan (left) and T-GEN (right) in intelligence GWAS data. Each dot represents a gene. The x-axis refers to the $-\log 10$ (p-value) indicating the TWAS significance level calculated by MAAT, and the y-axis refers to the $-\log 10$ (p-value) indicating the TWAS significance level calculated by PrediXcan (left) or T-GEN (right). The purple dashed line corresponds to the p-value cutoff of $5e-6$. Genes not reaching the significance level in both methods are represented with gray dots, genes reaching the significance level in MAAT while not reaching the significance level in other methods are represented with red dots, genes reaching the significance level in both methods are represented with cyan dots.

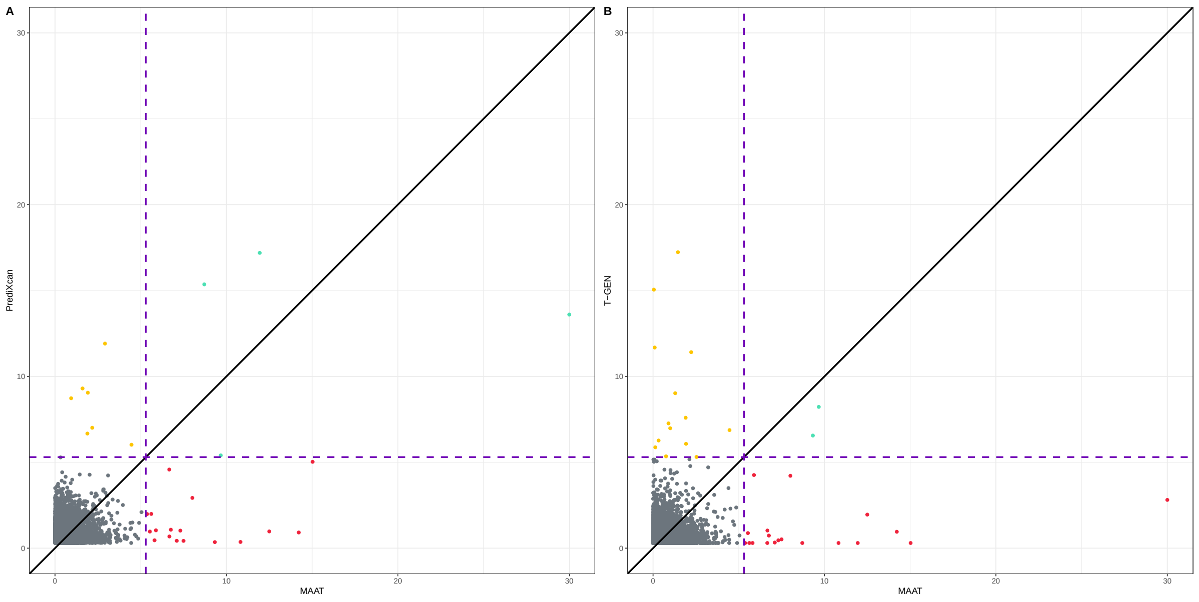

Fig. S28. Scatterplot of p-values for gene-trait associations comparing MAAT with PrediXcan (left) and T-GEN (right) in Parkinson's disease GWAS data. Each dot represents a gene. The x-axis refers to the $-\log 10$ (p-value) indicating the TWAS significance level calculated by MAAT, and the y-axis refers to the $-\log 10$ (p-value) indicating the TWAS significance level calculated by PrediXcan (left) or T-GEN (right). The purple dashed line corresponds to the p-value cutoff of $5e-6$. Genes not reaching the significance level in both methods are represented with gray dots, genes reaching the significance level in MAAT while not reaching the significance level in other methods are represented with red dots, genes reaching the significance level in both methods are represented with cyan dots.

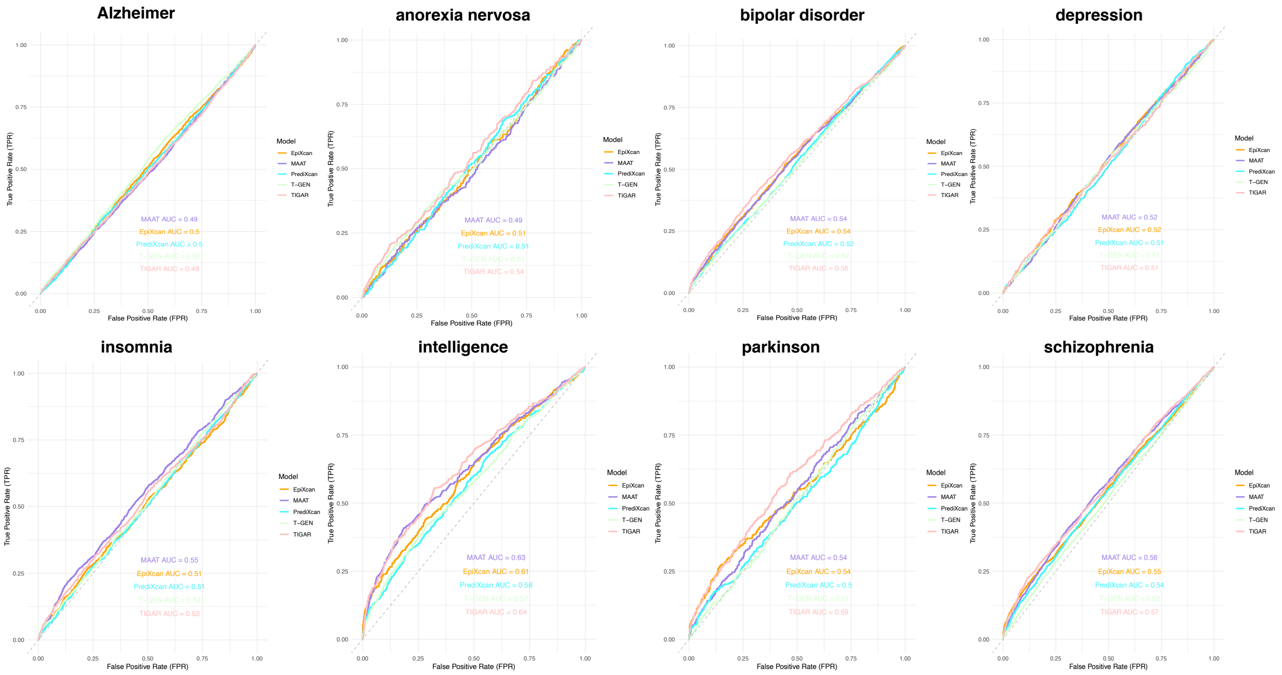

Fig. S29. Receiver operating characteristic (ROC) curve for the Online Mendelian Inheritance in Man (OMIM) and NHGRI-EBI GWAS catalog silver standard. Each subfigure represents an ROC analysis for a trait across five methods. The combination of OMIM database and NHGRI-EBI GWAS catalog database are set as silver standard. The x-axis refers to false positive rate, the y-axis refers to true positive rate. The area under the curve (AUC) value for each method is also given.

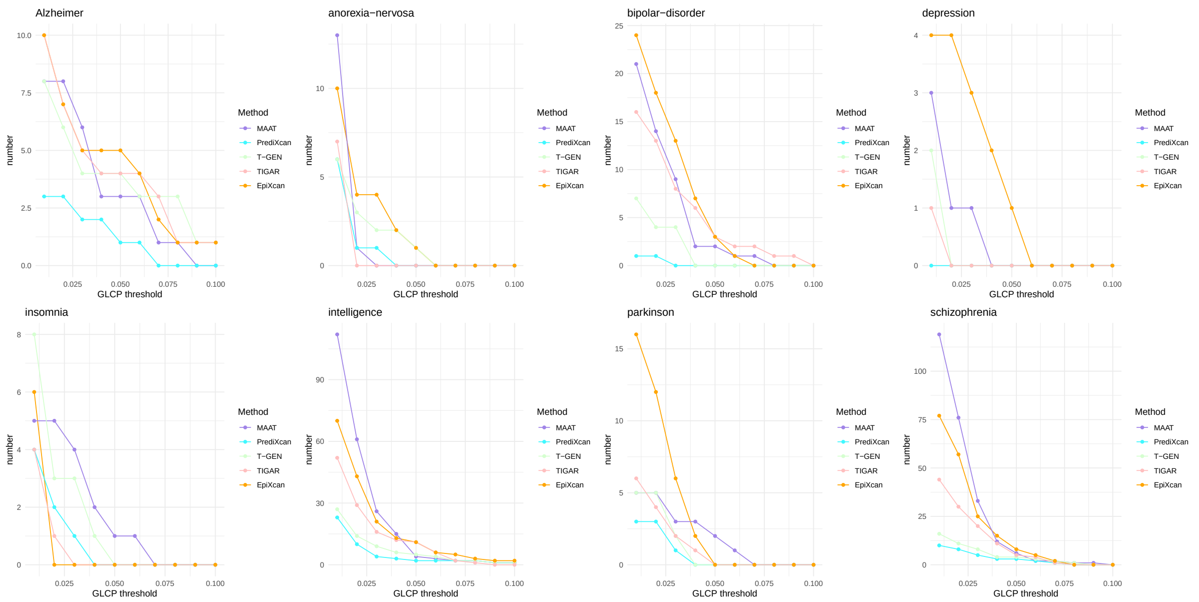

Fig. S30. The number of conceptual replications between ENLOC and five different TWAS methods. The x-axis represents different GLCP thresholds of ENLOC, the y-axis is the number of overlapping genes between TWAS significant genes and gene sets with GLCP greater than a specific threshold.

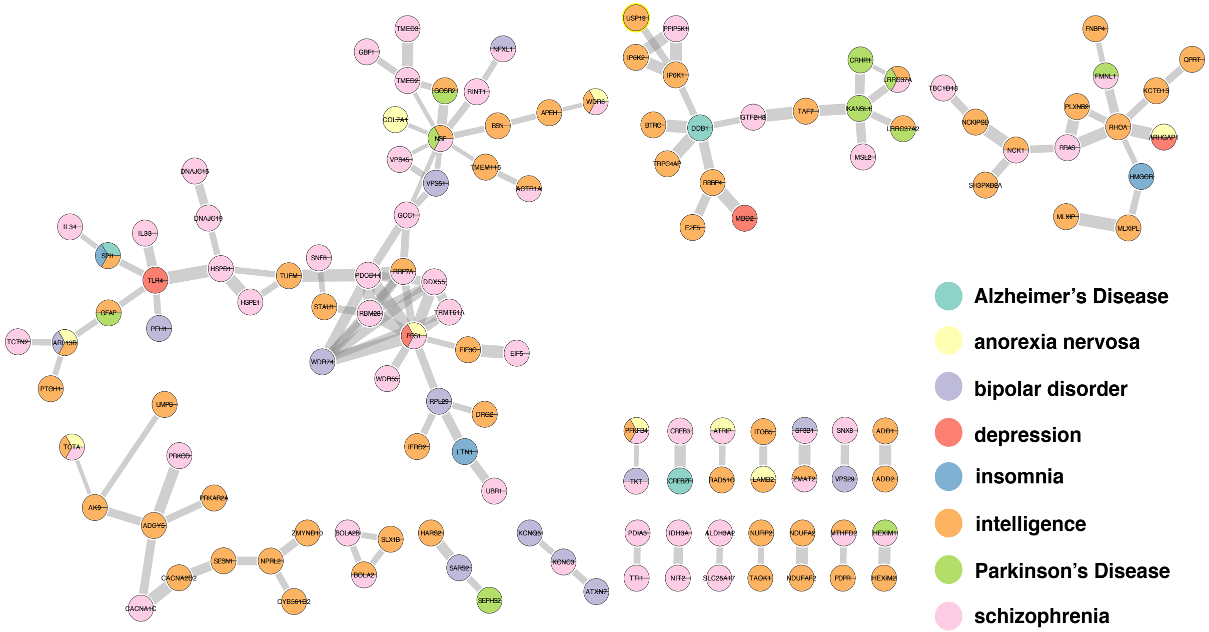

Fig. S31. STRING protein-protein network for TWAS significant genes identified by MAAT in eight psychiatric traits. Each circle represents a TWAS significant gene selected by MAAT (p-value$< 5 \times 10^{-6}$). Different colors for each gene represent the psychiatric traits in which they play a key role. The pie chart is adpoted to display genes which play functions in multiple traits. The thickness of the lines connecting the genes represents the strength of gene-gene interactions.

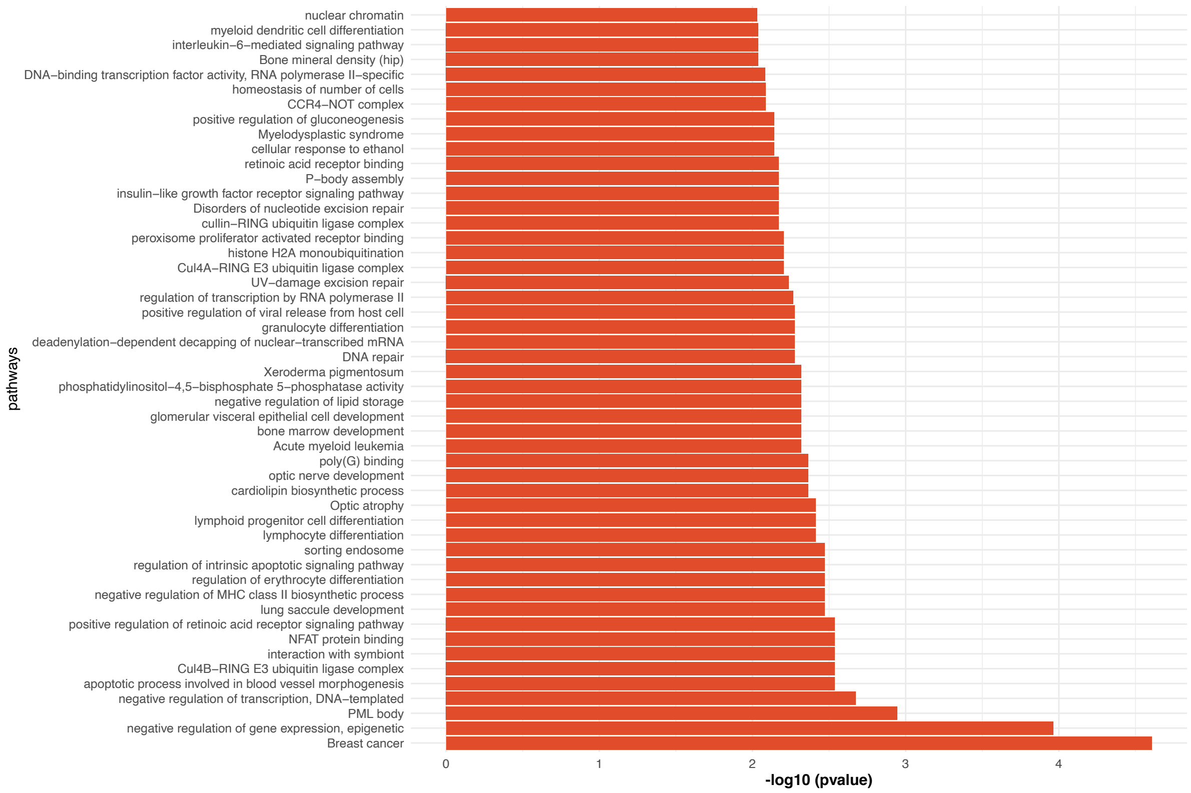

Fig. S32. Top 50 enriched pathways in Alzheimer's disease. For Alzheimer's disease, pathway enrichment analysis is performed in the gene set with high significance level (p-value$< 5e-6$) calculated by MAAT. Top 50 enriched pathways (adjusted p-value< 0.05) are listed. Each row represents a significantly enriched pathway in Alzheimer's disease. The x-axis is the $-\log 10$ (p-value) of the enrichment level.

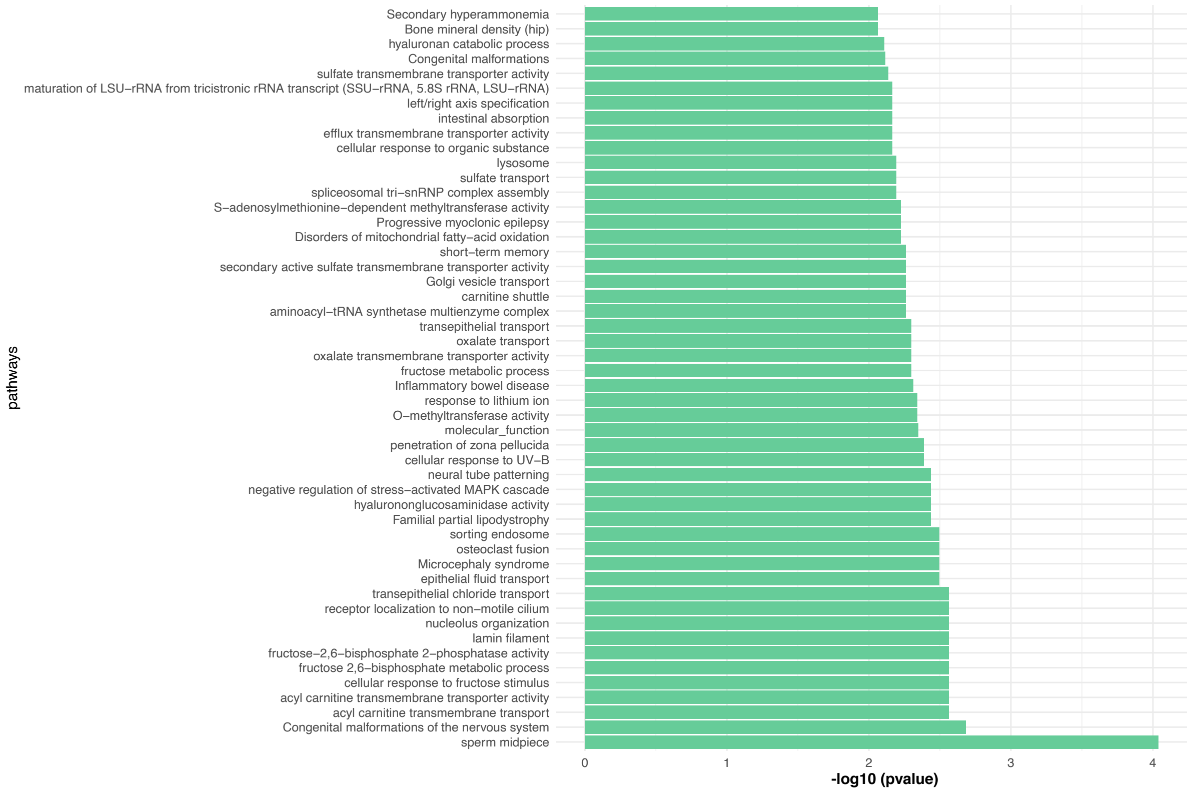

Fig. S33. Top 50 enriched pathways in anorexia nervosa. For anorexia nervosa, pathway enrichment analysis is performed in the gene set with high significance level (p-value$< 5e-6$) calculated by MAAT. Top 50 enriched pathways (adjusted p-value< 0.05) are listed. Each row represents a significantly enriched pathway in anorexia nervosa. The x-axis is the $-\log 10$ (p-value) of the enrichment level.

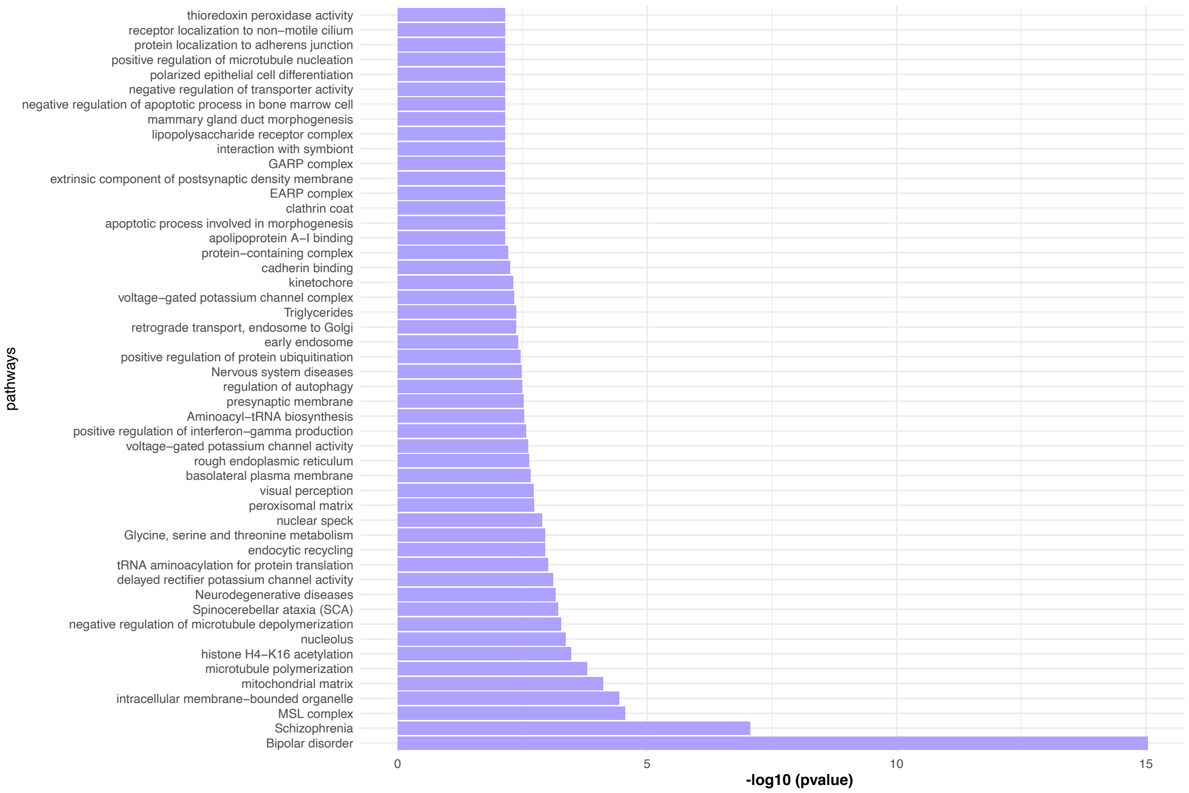

Fig. S34. Top 50 enriched pathways in bipolar disorder. For bipolar disorder, pathway enrichment analysis is performed in the gene set with high significance level (p-value$< 5e-6$) calculated by MAAT. Top 50 enriched pathways (adjusted p-value< 0.05) are listed. Each row represents a significantly enriched pathway in bipolar disorder. The x-axis is the $-\log 10$ (p-value) of the enrichment level.

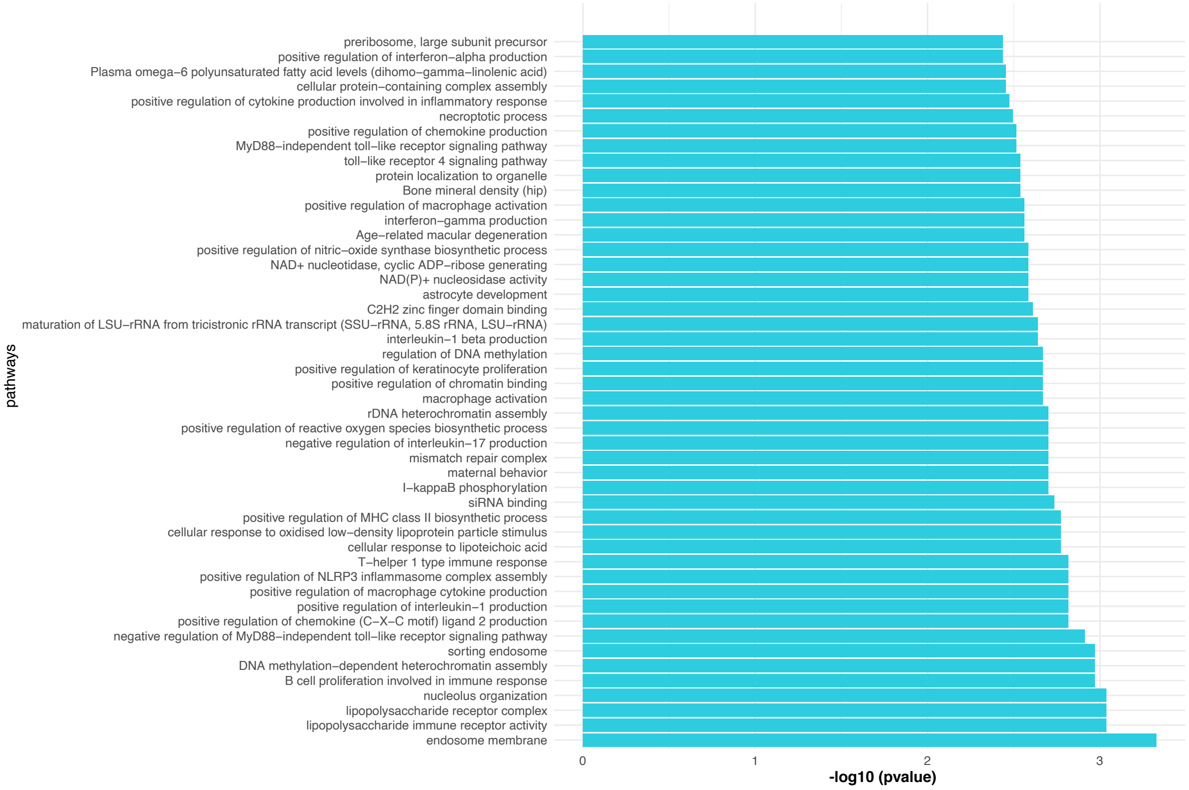

Fig. S35. Top 50 enriched pathways in depression. For depression, pathway enrichment analysis is performed in the gene set with high significance level (p-value$< 5e-6$) calculated by MAAT. Top 50 enriched pathways (adjusted p-value< 0.05) are listed. Each row represents a significantly enriched pathway in depression. The x-axis is the $-\log 10$ (p-value) of the enrichment level.

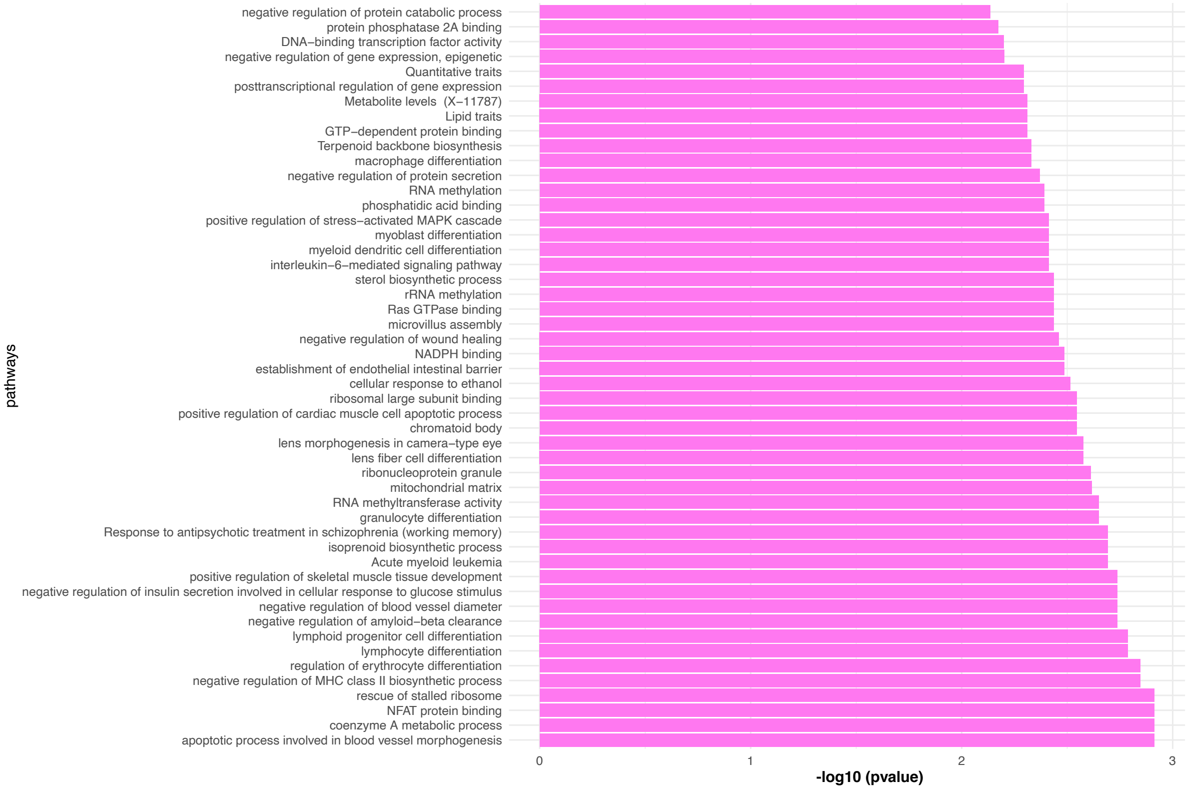

Fig. S36. Top 50 enriched pathways in insomnia. For insomnia, pathway enrichment analysis is performed in the gene set with high significance level (p-value$<5e-6$) calculated by MAAT. Top 50 enriched pathways (adjusted p-value<0.05) are listed. Each row represents a significantly enriched pathway in insomnia. The x-axis is the $-\log 10$ (p-value) of the enrichment level.

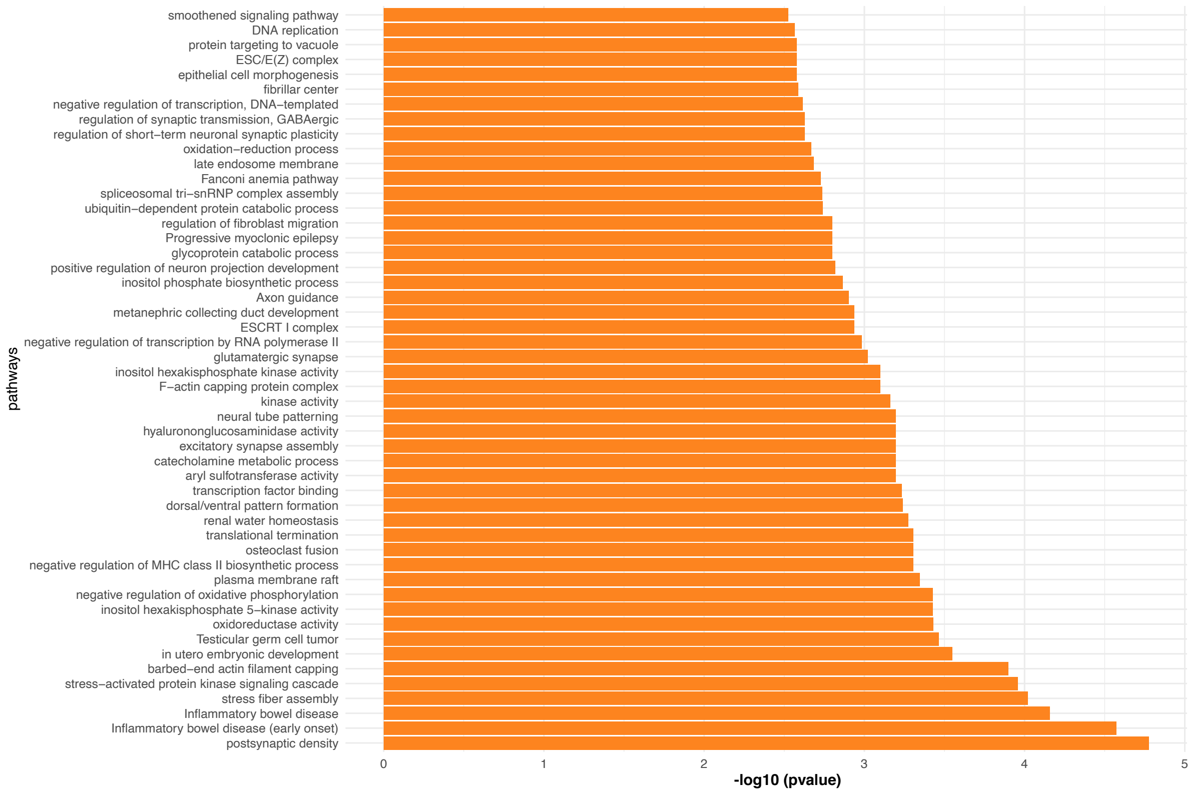

Fig. S37. Top 50 enriched pathways in intelligence. For intelligence, pathway enrichment analysis is performed in the gene set with high significance level (p-value$<5e-6$) calculated by MAAT. Top 50 enriched pathways (adjusted p-value<0.05) are listed. Each row represents a significantly enriched pathway in intelligence. The x-axis is the $-\log 10$ (p-value) of the enrichment level.

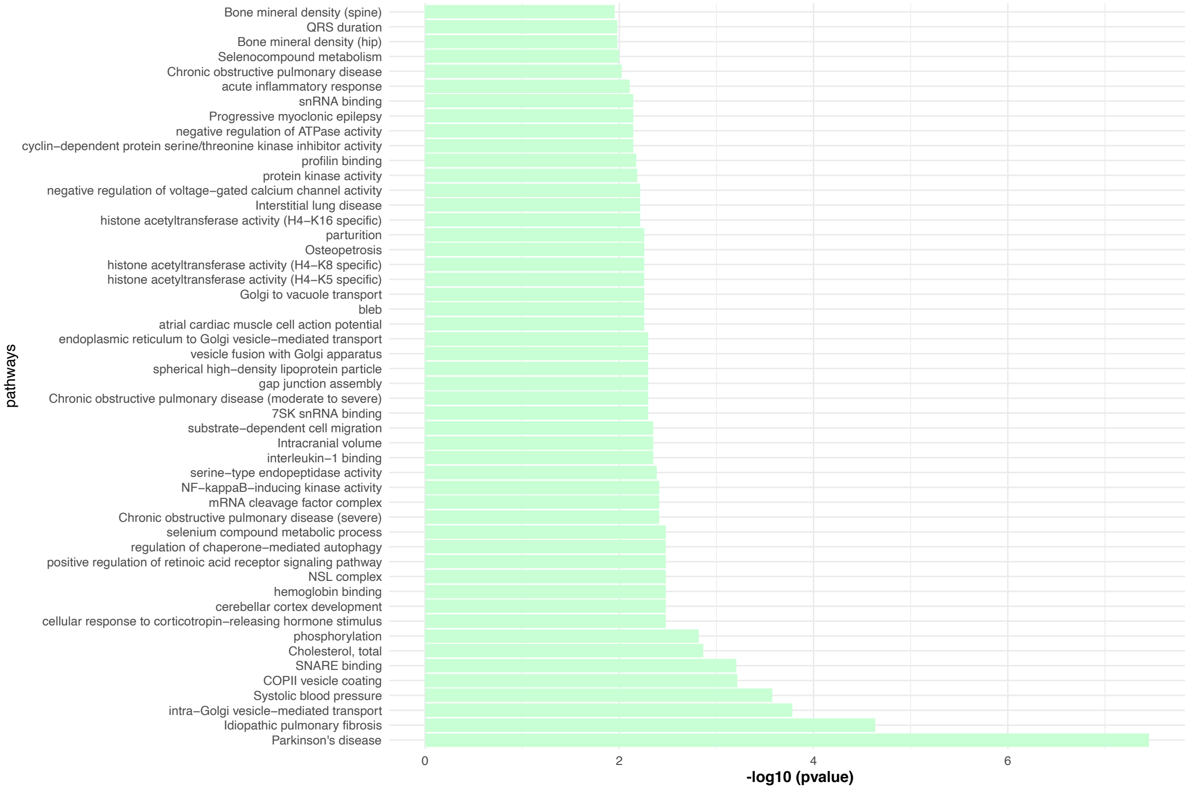

Fig. S38. Top 50 enriched pathways in Parkinson's disease. For Parkinson's disease, pathway enrichment analysis is performed in the gene set with high significance level (p-value$< 5e-6$) calculated by MAAT. Top 50 enriched pathways (adjusted p-value< 0.05) are listed. Each row represents a significantly enriched pathway in Parkinson's disease. The x-axis is the $-\log 10$ (p-value) of the enrichment level.

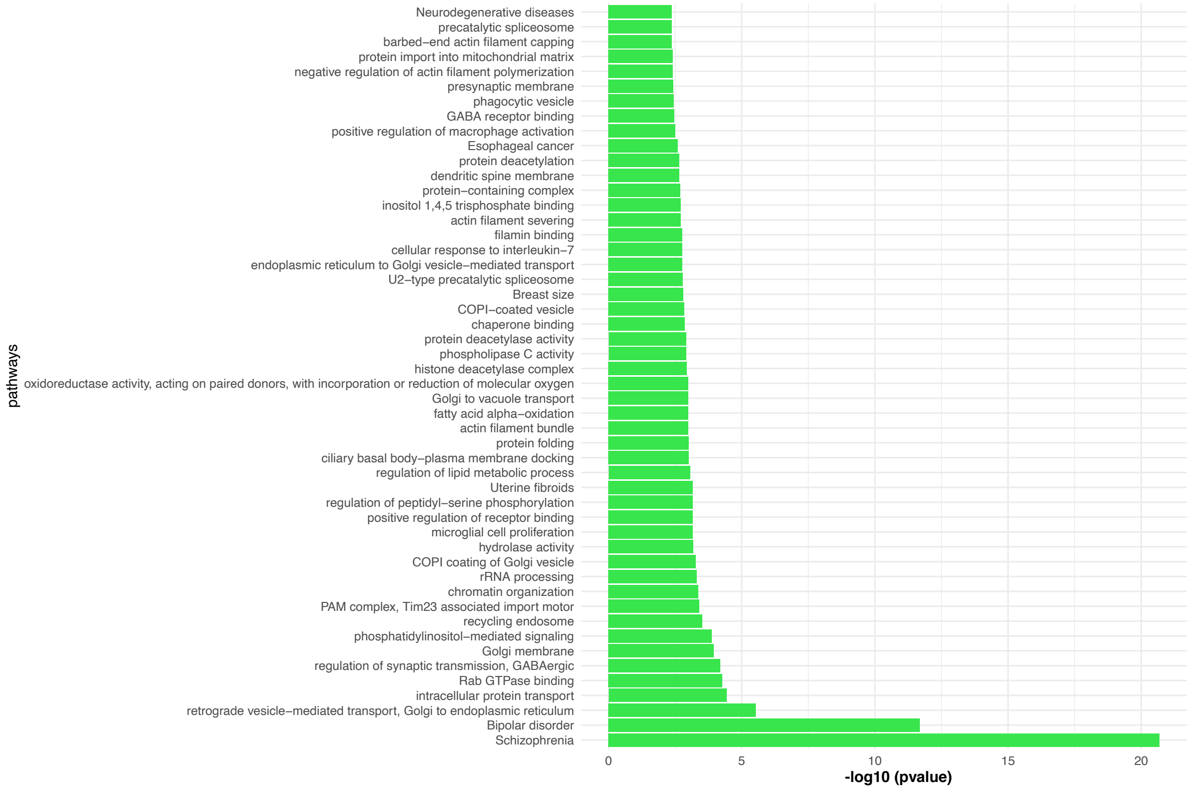

Fig. S39. Top 50 enriched pathways in schizophrenia. For schizophrenia, pathway enrichment analysis is performed in the gene set with high significance level (p-value$< 5e-6$) calculated by MAAT. Top 50 enriched pathways (adjusted p-value< 0.05) are listed. Each row represents a significantly enriched pathway in schizophrenia. The x-axis is the $-\log 10$ (p-value) of the enrichment level.

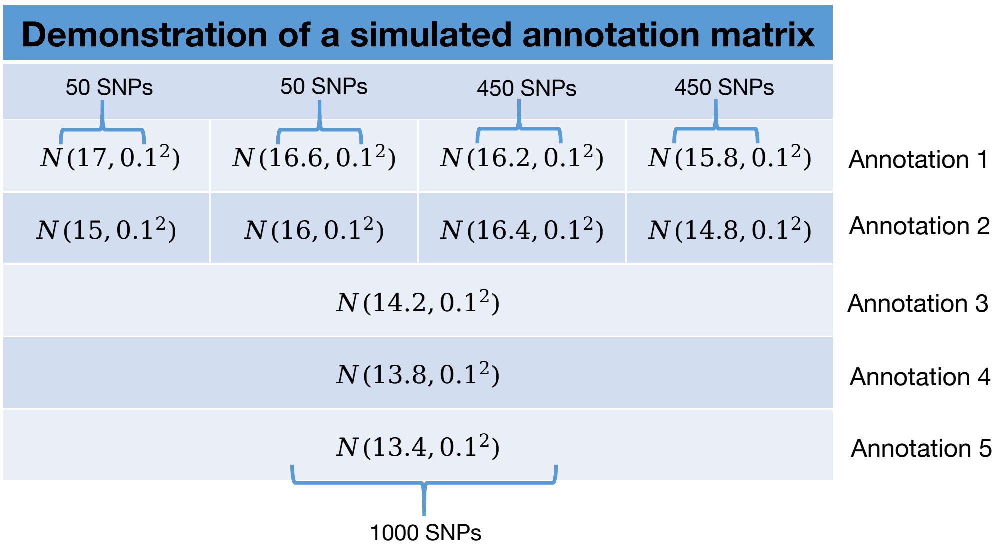

Fig. S40. A demonstration of the simulated annotation matrix. The annotation matrix $\boldsymbol{W} \in \mathbb{R}^{5\times 1000}$, with five annotations assigned to each cis-SNP. The first two rows represent the informative annotations, and the last three rows represent the non-informative annotations. When $p_{cs} = 0.1$, the 100 causal SNPs and 900 non-causal SNPs are both divided into two equal groups. Within each subgroup, the cis-SNPs' annotation score are sampled from the same normal distribution for each informative annotation. For the non-informative annotation, the annotation score for all the 1000 cis-SNPs are sampled from the same normal distribution.

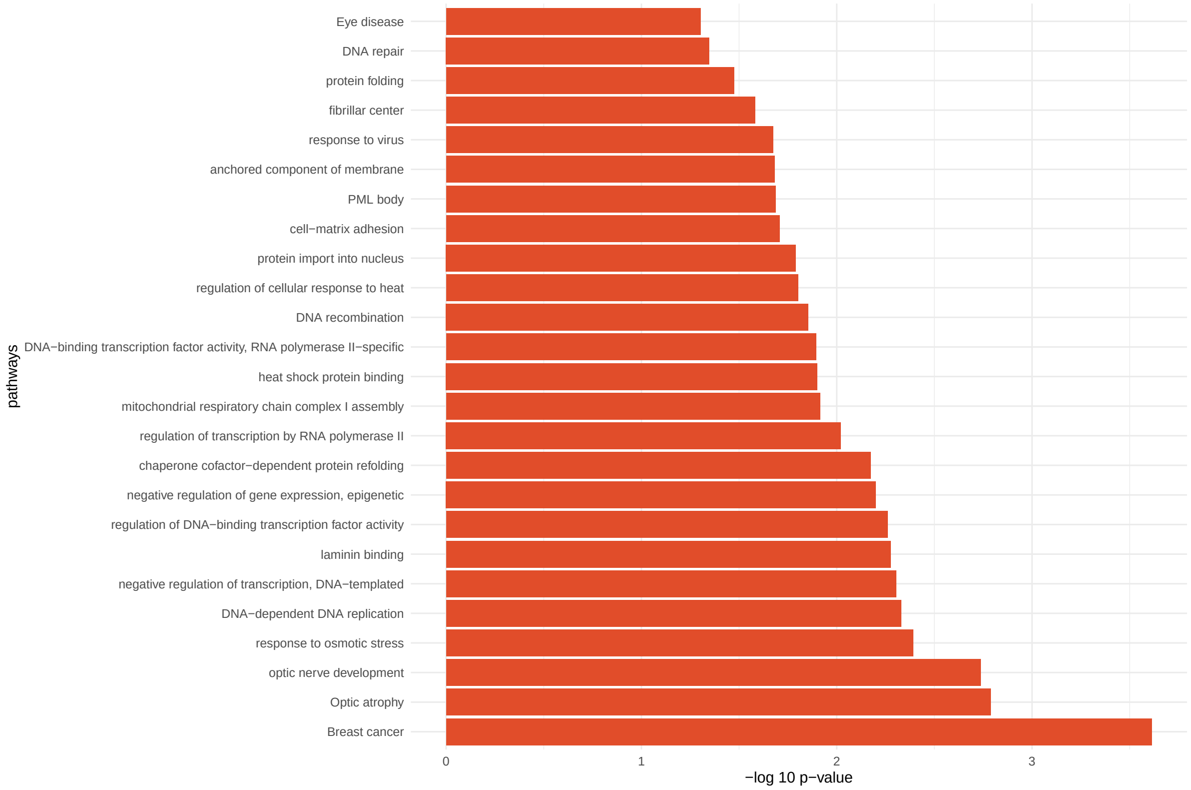

Fig. S41. Enriched pathways in Alzheimer's disease after colocalization analysis. For Alzheimer's disease, pathway enrichment analysis is performed in the overlapping set between TWAS significant genes and the gene set with GLCP larger than 0.001. Significantly enriched pathways (adjusted p-value< 0.05) are listed. Each row represents a significantly enriched pathway in Alzheimer's disease. The x-axis is the $-\log 10$ (p-value) of the enrichment level.

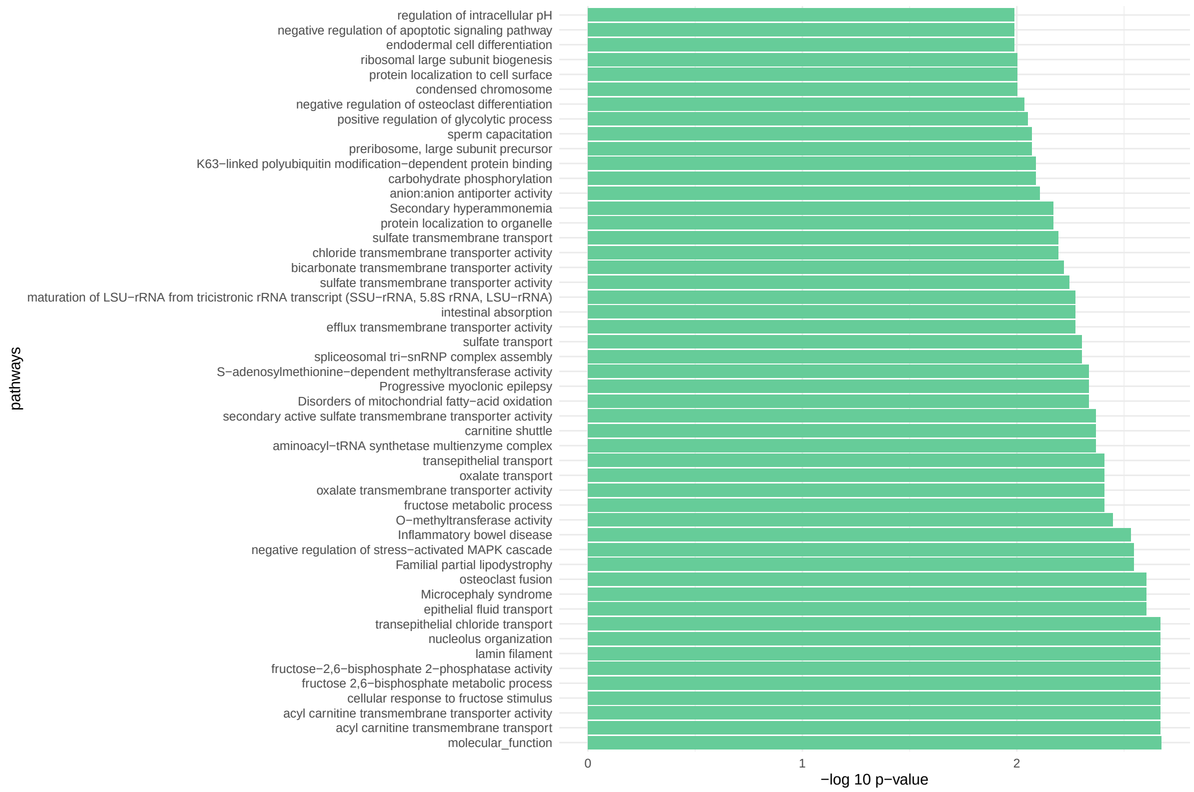

Fig. S42. Top 50 enriched pathways in anorexia nervosa after colocalization analysis. For anorexia nervosa, pathway enrichment analysis is performed in the overlapping set between TWAS significant genes and the gene set with GLCP larger than 0.001. Top 50 enriched pathways (adjusted p-value< 0.05) are listed. Each row represents a significantly enriched pathway in anorexia nervosa. The x-axis is the $-\log 10$ (p-value) of the enrichment level.

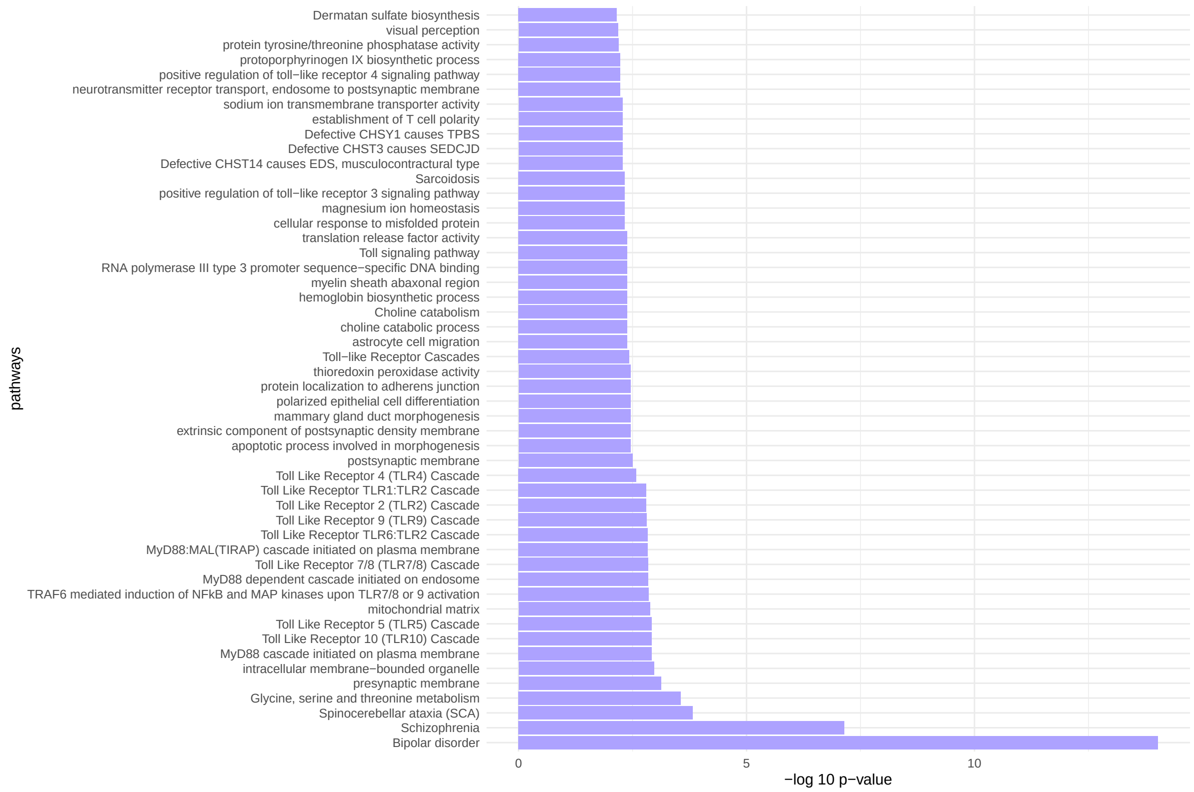

Fig. S43. Top 50 enriched pathways in bipolar disorder after colocalization analysis. For bipolar disorder, pathway enrichment analysis is performed in the overlapping set between TWAS significant genes and the gene set with GLCP larger than 0.001. Top 50 enriched pathways (adjusted p-value< 0.05) are listed. Each row represents a significantly enriched pathway in bipolar disorder. The x-axis is the $-\log 10$ (p-value) of the enrichment level.

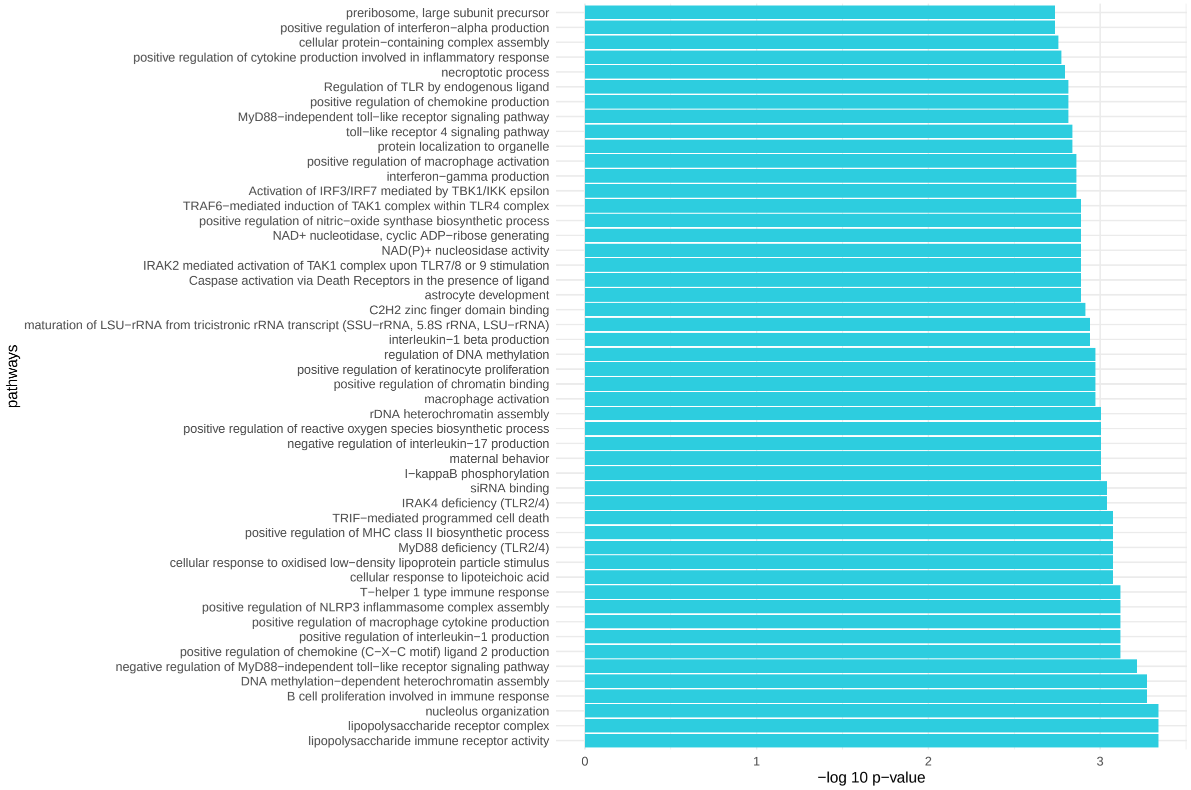

Fig. S44. Top 50 enriched pathways in depression after colocalization analysis. For depression, pathway enrichment analysis is performed in the overlapping set between TWAS significant genes and the gene set with GLCP larger than 0.001. Top 50 enriched pathways (adjusted p-value< 0.05) are listed. Each row represents a significantly enriched pathway in depression. The x-axis is the $-\log 10$ (p-value) of the enrichment level.

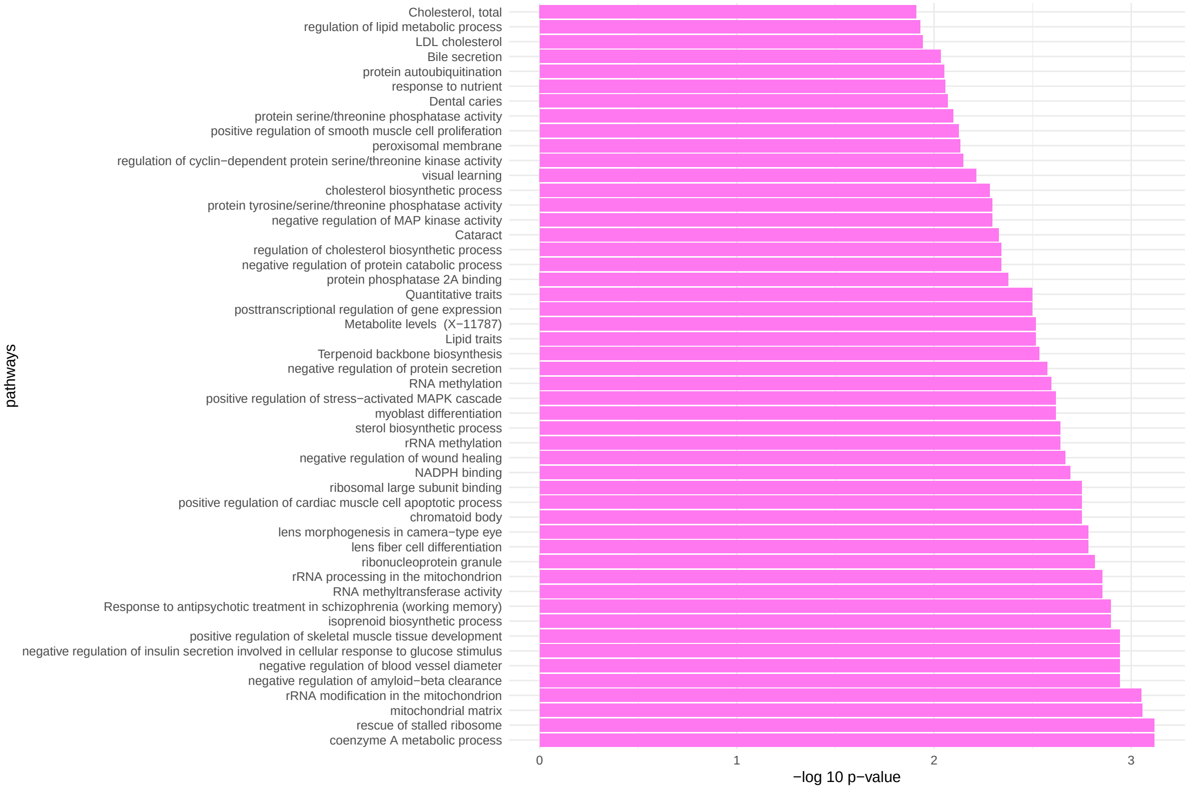

Fig. S45. Top 50 enriched pathways in insomnia after colocalization analysis. For insomnia, pathway enrichment analysis is performed in the overlapping set between TWAS significant genes and the gene set with GLCP larger than 0.001. Top 50 enriched pathways (adjusted p-value< 0.05) are listed. Each row represents a significantly enriched pathway in insomnia. The x-axis is the $-\log 10$ (p-value) of the enrichment level.

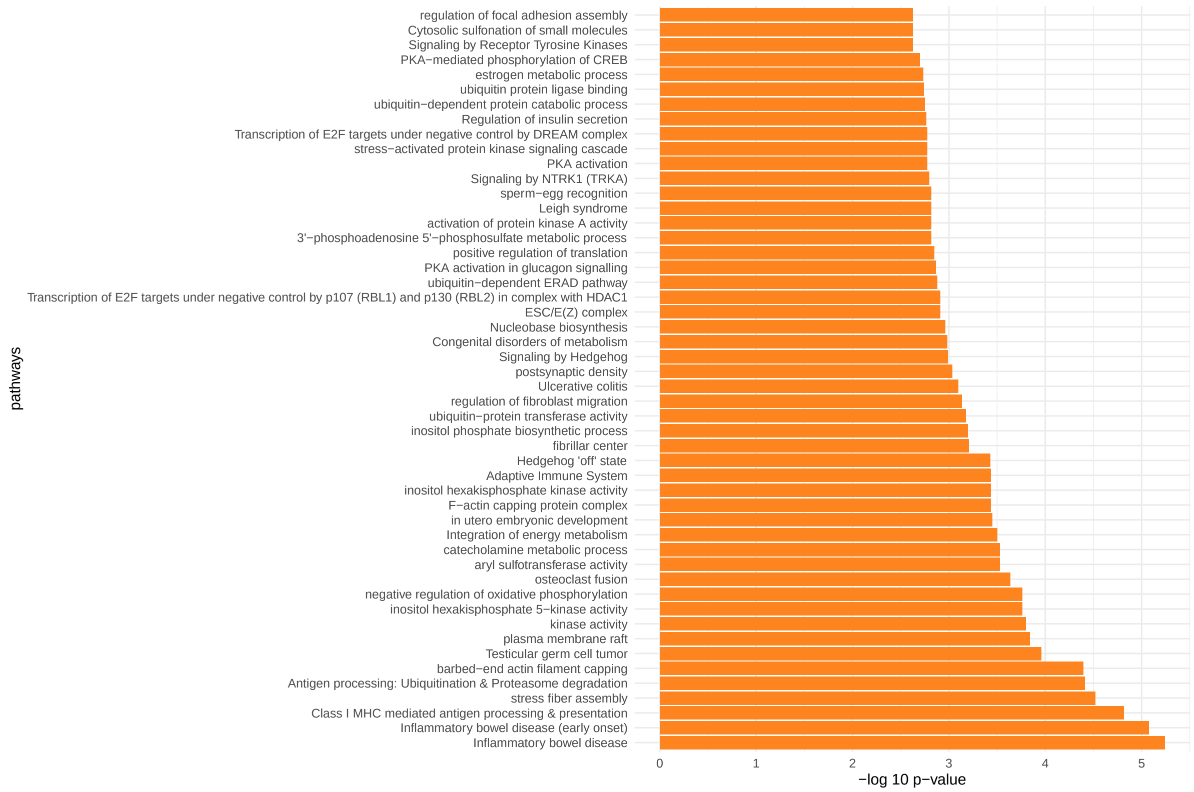

Fig. S46. Top 50 enriched pathways in intelligence after colocalization analysis. For intelligence, pathway enrichment analysis is performed in the overlapping set between TWAS significant genes and the gene set with GLCP larger than 0.001. Top 50 enriched pathways (adjusted p-value< 0.05) are listed. Each row represents a significantly enriched pathway in intelligence. The x-axis is the $-\log 10$ (p-value) of the enrichment level.

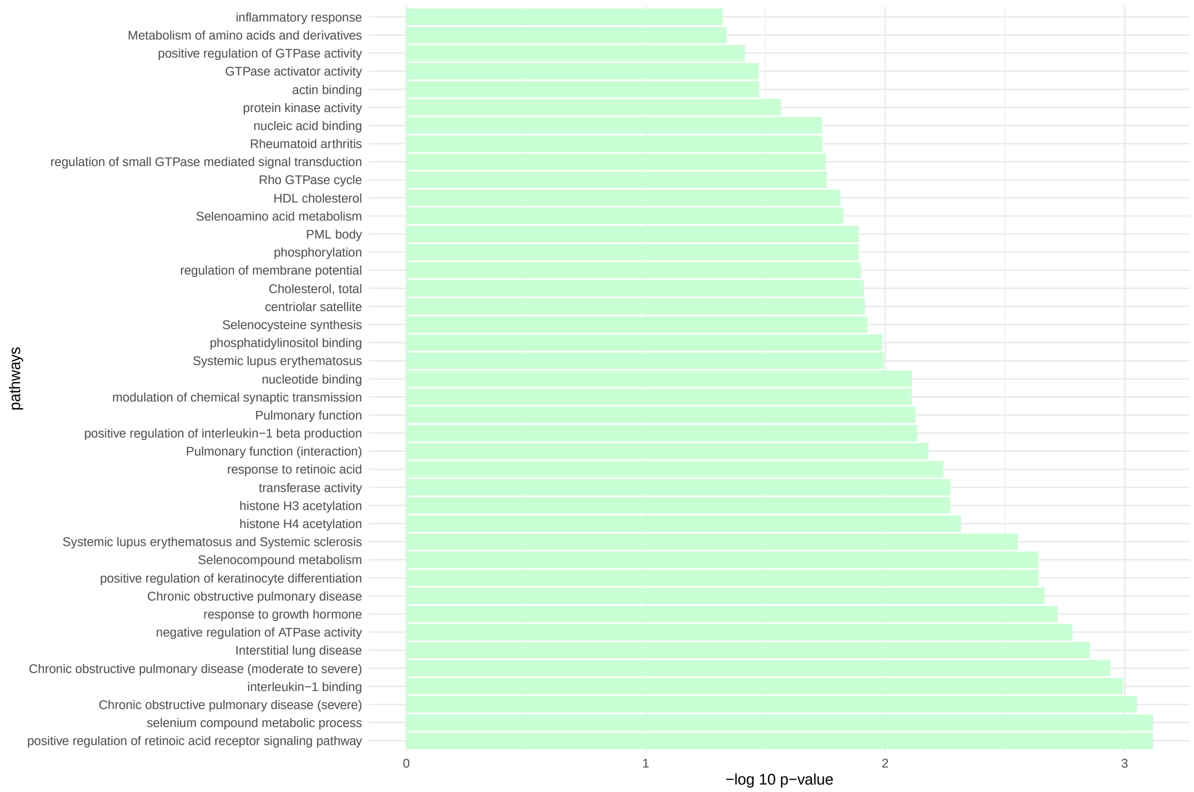

Fig. S47. Enriched pathways in Parkinson's disease after colocalization analysis. For Parkinson's disease, pathway enrichment analysis is performed in the overlapping set between TWAS significant genes and the gene set with GLCP larger than 0.001. Significantly enriched pathways (adjusted p-value<0.05) are listed. Each row represents a significantly enriched pathway in Parkinson's disease. The x-axis is the $-\log 10$ (p-value) of the enrichment level.

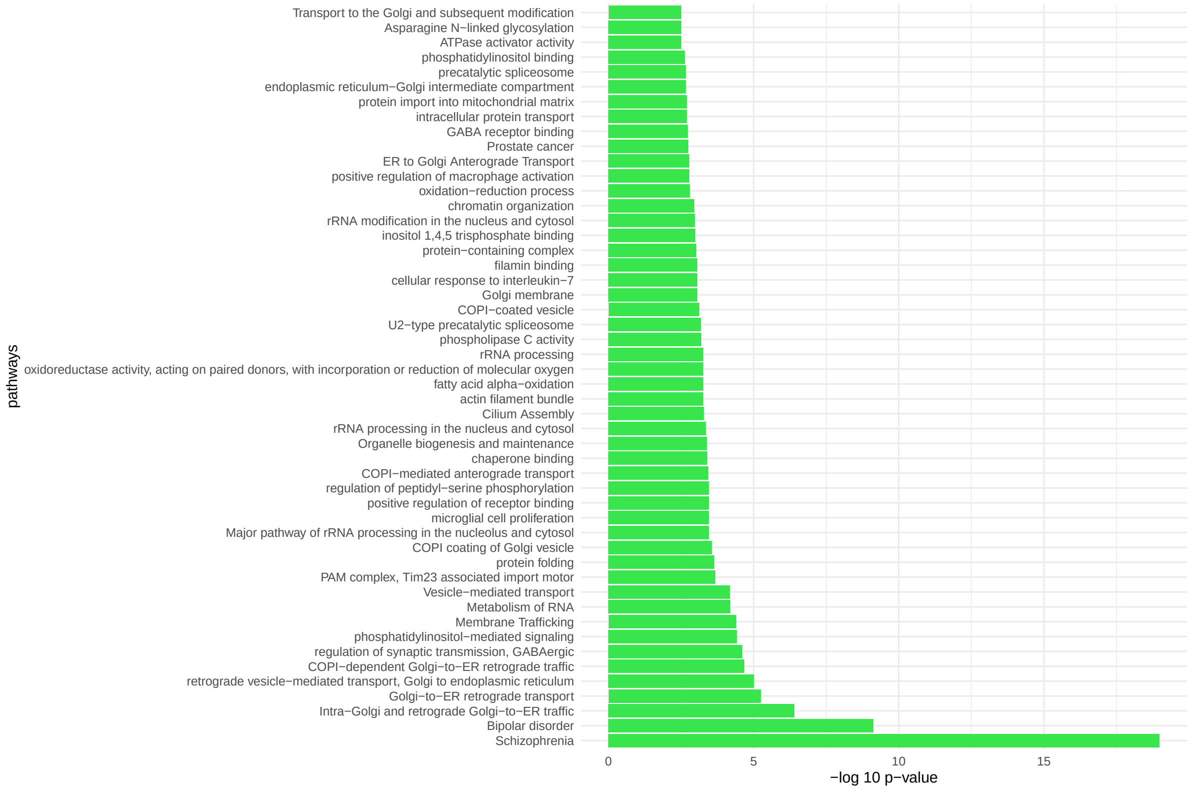

Fig. S48. Top 50 enriched pathways in schizophrenia after colocalization analysis. For schizophrenia, pathway enrichment analysis is performed in the overlapping set between TWAS significant genes and the gene set with GLCP larger than 0.001. Top 50 enriched pathways (adjusted p-value< 0.05) are listed. Each row represents a significantly enriched pathway in schizophrenia. The x-axis is the $-\log 10$ (p-value) of the enrichment level.
